# A systematic review and meta-analysis of the first decade of compositional data analyses of 24-hour movement behaviours, health, and well-being in school-aged children

**DOI:** 10.1186/s44167-025-00076-w

**Published:** 2025-03-27

**Authors:** Nicholas Kuzik, Markus J. Duncan, Natalie Beshara, Matthew MacDonald, Diego Augusto Santos Silva, Mark S. Tremblay

**Affiliations:** 1 Healthy Active Living and Obesity Research Group, Children’s Hospital of Eastern Ontario Research Institute, 401 Smyth Road, Ottawa, ON K1H 8L1 Canada; 2 ParticipACTION, 4 New Street, Toronto, ON M5R 1P6 Canada; 3 National University of Ireland, University Road, Galway, H91 TK33 Ireland; 4 Federal University of Santa Catarina. Florianopolis, Santa Catarina, 88040900 Brazil; 5 Department of Pediatrics, University of Ottawa, 401 Smyth Road, Ottawa, ON K1H 8L1 Canada

**Keywords:** Movement behaviours, Compositional data analysis, Sleep, Sedentary behaviour, Physical activity

## Abstract

**Introduction:**

Movement behaviours (e.g., sleep, sedentary behaviour, light physical activity [LPA], moderate to vigorous physical activity [MVPA]) are associated with numerous health and well-being outcomes. Compositional data analyses (CoDA) accounts for the interdependent nature of movement behaviours. This systematic review and meta-analysis provides a timely synthesis of the first decade of CoDA research examining the association between movement behaviours, health, and well-being in school-aged children.

**Methods:**

Databases were systematically searched for peer-reviewed studies examining CoDA associations between movement behaviours and health or well-being in school-aged children (5.0-17.9 years). All health and well-being outcomes were eligible for inclusion, as were all methods of reporting CoDA results. Where possible meta-analyses were conducted.

**Results:**

Twenty-six studies were included in the review. Sample sizes ranged from 88 − 5,828 (median = 387) participants and the mean ages ranged from 8 to 16 years. Regression parameters (*k*_studies_=16) were the most common method of reporting results, followed by substitution effects (*k*_studies_=12), optimal compositions (*k*_studies_=3), and movement behaviour clusters (*k*_studies_ =1). Weighted compositional means of movement behaviours were calculated (e.g., 49.8 min/day of MVPA). For regression analyses, results were generally null, though some favourable trends were observed for MVPA and unfavourable trends for LPA and sedentary behaviour within individual health and well-being outcomes categories. Meta-analyses of substitutions supported the benefits of MVPA, with the risks of reducing MVPA for other movement behaviours being double the magnitude compared to the benefits of adding MVPA.

**Discussion:**

The most consistent conclusions within this review align with previous reviews that support the benefits of MVPA. Further, some evidence supported 24-hour movement behaviour guideline recommendations of increasing sleep and decreasing sedentary behaviour. This review also quantified not only the need to promote MVPA, but perhaps more importantly the urgency needed to preserve the limited MVPA children currently accumulate. Findings reinforce the “more/less is better” messages for movement behaviours, but do not allow us to recommend more specific balances of movement behaviours. As CoDA of movement behaviours progresses and accumulates further research, the methods and discussion points within the current review can aide future meta-analyses aimed at advancing the precision health guidance needed for optimizing children’s health and well-being.

**Supplementary Information:**

The online version contains supplementary material available at 10.1186/s44167-025-00076-w.

## Introduction

Movement behaviours, including sleep, sedentary behaviours, and physical activity in children and adolescents are important for health and well-being across the lifespan. Accumulating sufficient amounts of sleep and physical activity while minimizing sedentary behaviours is associated with numerous physical and mental health benefits during childhood and adolescence [1]. Moreover, health and health behaviours in childhood and adolescence also have important ramifications into adulthood. Adolescence marks the peak onset of mental illnesses, contributing significantly to chronic disability throughout life [2, 3]. Children with a positive sense of well-being are more likely to attend school regularly, perform better academically, and engage in fewer risk-taking behaviours—all of which are important for success later in life [4–6]. Additionally, adhering to movement behaviour recommendations in childhood often tracks into adulthood, where these behaviours continue to positively influence health and well-being outcomes [7–10]. However, movement behaviour patterns for most children and adolescents are currently insufficient and risk missing the opportunity for improving health and well-being across the lifespan. For instance, pooled report card grades across 57 countries in the Global Matrix 4.0 indicated children and adolescents received a D grade for overall physical activity (27–33% meeting the recommended ≥ 60 min/day of moderate to vigorous physical activity [MVPA]) and a D + for sedentary behaviour (34–39% meeting the recommended ≤ 2 h/day of recreational screen time) [11, 12].

However, much of the existing evidence examines the associations between movement behaviours and health in a linear or independent manner, often resulting in a generic “more is better” conclusion for sleep and MVPA, and the inverse for sedentary behaviour. While this may vaguely outline the true relationship between movement behaviours and health (especially when most individuals are accruing MVPA below recommended levels), these analyses ignore the co-dependent nature of 24-hour time use, where changes in one movement behaviour necessitate compensatory changes in one or more other behaviours. Furthermore, the interdependent relationships between these behaviours suggest that the benefits of exchanging one behaviour for another may vary depending on how much time is spent in other behaviours. For example, the benefits of exchanging sedentary behaviour for more MVPA may depend on how much sleep an individual is accumulating. Additionally, diminishing returns, or even adverse effects, may arise when time spent in one behaviour exceeds certain thresholds, even when replacing behaviours traditionally considered less beneficial. Fundamentally, movement behaviours in the context of 24-hour time use represent an optimization problem where optimal combinations of behaviour may lead to the greatest benefit [13].

Compositional data analysis (CoDA) provides a method for addressing this optimization issue for a constrained 24-hour time period by treating data on time spent in movement behaviours as relative information [14, 15]. Such relative information thus becomes inherently co-dependent as the proportion of time spent in any one movement behaviour is contextualized based on the amount of time spent in all remaining behaviours. Regression models based on CoDA can address the optimization issue of 24-hour time use by predicting optimal ratios of movement behaviours associated with the best and worst health outcomes [16–18]. Thus, continued efforts are essential to better understand the optimal distribution of movement behaviours for health and well-being.

Systematic reviews examining CoDA associations among movement behaviours and health outcomes in children and youth have generally found that more MVPA is favourable for many health outcomes [1, 19–21]. Further, there is less consistent evidence suggesting that on top of increasing MVPA, reducing sedentary behaviour and increasing sleep are beneficial for health in children and adolescents [1, 19–21]. While the general call for increasing MVPA and sleep while decreasing sedentary behaviours would likely be impactful for global health, there is less direction for how much to increase or decrease any of these behaviours. A meta-analysis focused on compositional movement behaviours could offer important insights into these dynamics, providing more actionable and precise recommendations for public health. Despite the challenges involved in meta-analyzing compositional movement behaviours (e.g., lack of standardized reporting of CoDA results), this review undertakes this critical task while also providing a comprehensive summary of the existing literature on compositional movement behaviours and health and well-being in school-aged children.

### Review questions

#### Primary

- What is the optimal distribution of 24-hour movement behaviours for health and well-being in children and adolescents aged 5.00-17.99 years?

#### Secondary

- What are the relationships between compositional movement behaviour durations and patterns (e.g., bouts) for health and wellness in children and adolescents aged 5.00-17.99 years?
- What is the optimal distribution of 24-hour movement behaviours in the domains of physical, cognitive, and mental health in children and adolescents aged 5.00-17.99 years?
- What are significant covariates related to the optimal distribution of 24-hour movement behaviours for health and wellness in children and adolescents aged 5.00-17.99 years?

## Methods

### Registration and protocol

This review was registered with the International Prospective Register of Systematic Reviews (PROSPERO ID:42022378220) and followed the Preferred Reporting Items for Systematic Reviews and Meta-Analyses (PRISMA) guidelines [ [22]; Additional File 1].

### Eligibility criteria

#### Study design

Any study design was eligible regardless of whether data were observational or interventional in nature. Grey literature (e.g., conference papers, dissertations), reviews and commentaries were excluded. Studies were required to be published in English.

#### Population

The population age range was selected to align with 24-Hour Movement Guidelines for Children and Youth developed for individuals 5 to 17 years of age [12]. Studies were included if the mean age of the sample was within this range for at least one time point, where movement behaviours were measured as an exposure for a health outcome. When studies reported age-stratified data where one or more groups met these criteria, the stratified results were also eligible for inclusion even if the mean age of the overall study sample was outside the defined range. Studies were limited to apparently healthy samples (i.e., general populations, including those with overweight/obesity); studies examining only children and youth diagnosed with a specific medical condition were excluded.

#### Intervention/exposure

The exposure was the composition of movement behaviours over the 24-hour day. Movement behaviours could be device measured (e.g., accelerometer) or self- or proxy-reported (e.g., sleep log) and were defined based on a continuum of energy expenditure that includes sleep, sedentary behaviour and physical activity [23]. To be included, all studies were required to include some measure of (1) sleep, (2) sedentary behaviour, and (3) physical activity within the composition. Sub-categories within this continuum were permitted; for example, distinctions between varying intensities of physical activity (e.g., light, moderate, vigorous) or contextual distinctions (e.g., sedentary behaviour during or outside of school time). Studies that did not define time use within these broad movement behaviour categories were excluded. Studies that combined any of the three main continuum behaviours (e.g. combining sleep and sedentary behaviour) were also excluded. Studies were required to assess the exposure to movement behaviours using CoDA techniques.

Studies were also required to attempt to measure movement behaviours across the 24-hour day. No criteria were set for a minimum measurement amount required to be considered a full day (e.g., > 20 h of the day accounted for), however studies were excluded if they explicitly examined only movement behaviours occurring during a sub-component of the day (e.g., movement occurring only during the waking day or at school).

#### Comparison

Comparisons consisted of differences in 24-hour movement behaviour compositions both between- and within-subjects as appropriate to the study design.

#### Outcomes

All health and well-being outcomes were considered. A priori, these were anticipated to include mental well-being and mental illness indicators (e.g., resilience, disruptive behaviours, self-esteem, stress), cognitive indicators (e.g., academic achievement, spatial reasoning), physical health indicators (e.g., adiposity, fitness, cardiovascular disease risk factors), and health behaviours (e.g., gambling, substance abuse). Due to heterogeneity across the physical health indicators category, results were separated into the *post hoc* categories of adiposity indicators, cardiometabolic biomarkers, fitness, motor skills, and musculoskeletal growth. Additionally, an overall health and well-being category was created that included all health and well-being outcomes.

### Search strategy

The following electronic databases were searched on May 24, 2024: MEDLINE, EMBASE, PsycINFO, CINAHL, and SPORTDiscus (See Additional File 2). Articles needed to be peer-reviewed and in English. Articles published before January 1, 2014 were excluded since this predates the application of CoDA to movement behaviour research [24].

### Study selection

Bibliographic records were extracted and imported into the Zotero (Corporation for Digital Scholarship, Virginia, USA) reference management software package to remove duplicate references. In level 1 screening, titles and abstracts of potentially relevant articles were screened by 3 reviewers (MD, MM, & NK) using Covidence (Veritas Health Innovation, Melbourne, Australia). In level 2 screening, full-text copies of articles were obtained for those meeting initial screening. Three reviewers examined all full-text articles (MD, MM, & NK). Included articles needed to be accepted by two reviewers, with any discrepancies resolved with a discussion and consensus between the 2 reviewers.

### Data extraction

Google Sheets was used for data extraction. Data extractions were performed by three reviewers (NB, MD, or NK). After a reviewer extracted data for a study, a second reviewer verified the accuracy and fulsomeness of the extracted study data. Any discrepancies in extraction were flagged and resolved in discussion between the data extraction team.

Information was extracted regarding descriptive study characteristics (e.g., author, publication year, study design, country, sample size, age, sex), exposure, outcome, results, covariates, and confounders. Where multiple models were reported (e.g., bivariate and adjusted models), results were extracted from the bivariate and most fully adjusted model. Findings were determined to be statistically significant if *p* < 0.05 or a significant 95% confidence interval was reported. Reviewers were not blinded to the authors or journals when extracting data.

### Study quality assessment

The quality of primary research contributing to each health and well-being outcome category was assessed using the Joanna Briggs Institute (JBI) critical appraisal checklist [25, 26]. Study quality was assessed by one reviewer (NB, MD, or NK) and verified by one of the other reviewers, with discrepancies resolved through discussion. In an effort to include all available evidence, study quality did not influence eligibility for inclusion. Levels of quality were classified for each study and health and well-being outcome category, with more than 80% considered high quality, 41–80% considered moderate quality, and less than 41% considered low quality [27]. Mean study quality was calculated to summarize quality for each outcome category.

### Data syntheses

Based on the extracted data, most of the a priori primary and secondary research questions could not be addressed. Thus, a *post hoc* synthesis instead focused on the trends in results for health and well-being categories and overall health and well-being for the main extracted analysis categories. Specifically, the three main categories of results included: (1) regression coefficients indicating the significance of a single movement behaviour in relation to the remaining movement behaviour composition, (2) compositional substitution analyses attempting to determine the effect of substituting time spent in one movement behaviour for another, and (3) identifying optimal movement behaviour distributions or “Goldilocks days” analyses, where many substitutions were used in an attempt to find a range of movement behaviours associated with the optimal health or well-being outcomes. Where *k*_studies_>1 reported any of these three types of results, meta-synthesis of quantitative results was attempted. All other compositional analyses were narratively synthesized.

When studies presented multiple analyses, the main analysis was selected [i.e., Haszard, et al. [28] non-wear time reallocated to day-time components model used], and when only subgroups were presented [i.e., Dumuid, et al. [29], overall results not presented, only boys and girls separately] both subgroups were included. Values were reverse scored for outcomes where favourable associations are in the negative direction (i.e., BMI, waist circumference, waist to height ratio, body fat, fat mass index, C-reactive protein, triglycerides, insulin, blood pressure, strengths and difficulties score, 50-m run time).

#### Linear regression analyses

Results were classified as linear regression analyses when log-ratio transformed regression coefficients indicating the significance of a single movement behaviour in relation to the remaining movement behaviour composition were reported. Within movement behaviour research these regression coefficients are typically isometric log-ratio transformations done in a series called pivot coordinates, whereby the influence of individual movement behaviours in relation to the rest of the composition can be assessed. Considering pivot coordinates in isolation can determine the direction of results, but not the magnitude of results, summarizing the direction of findings was planned for studies reporting linear regression coefficients of movement behaviours and health. For instance, the number of findings in studies demonstrating that MVPA was favourable, null, and unfavourable for health in relation to the rest of the composition of movement behaviours were counted.

#### Compositional substitution analyses

For substitutions, a meta-analysis was conducted for each main substitution category (e.g., more MVPA, less sedentary) and across all health and well-being outcomes. The substitution effect is the mean difference when substituting one behaviour for another (e.g., one-for-one, one-for-all), so it was used to calculate within-group standardized mean differences by dividing the substitution effect by the baseline standard deviation of the outcome (when substitution standard deviations were not available). The mean difference value was also standardized to a 10-minute substitution, while assuming linearity. For instance, if a study examined 30-minute substitutions the mean difference was divided by 3 to achieve a 10-minute standardization. A 10-minute substitution was selected as the standardization value since it was the most common substitution value. When multiple substitutions of varying durations were reported for the same sets of movement behaviours, only the substitution values closest to 10 min were used. For example, Domingues, et al. [30] reported substitution increments of 75, 90, and 120 min/day, so the 75-minute substitutions were selected for meta-analysis. Additionally, Haszard, et al. [28] reported substitutions in both 10 min/day and 10% of the day increments, thus 10 min/day substitutions were selected for meta-analysis.

The standard error of the within-group standardized mean differences was calculated while assuming a correlation of 0.59 [31]. To account for unit-of-analysis errors, when multiple outcomes were reported in one study, they were aggregated using the R package *metafor* [32]. A high degree of heterogeneity was expected due to analyzing many different health outcomes and measures, so the random effects models were used. Thus, the standard mean difference and 95% confidence interval on overall health and well-being when substituting each main movement behaviour for one another was calculated while grouping studies based on cross-sectional and longitudinal findings.

#### Optimal movement behaviour distributions

The weighted mean and other descriptive values were planned for the optimal movement behaviour compositions identified across studies.

## Results

### Screening and study characteristics

**Fig. 1:**
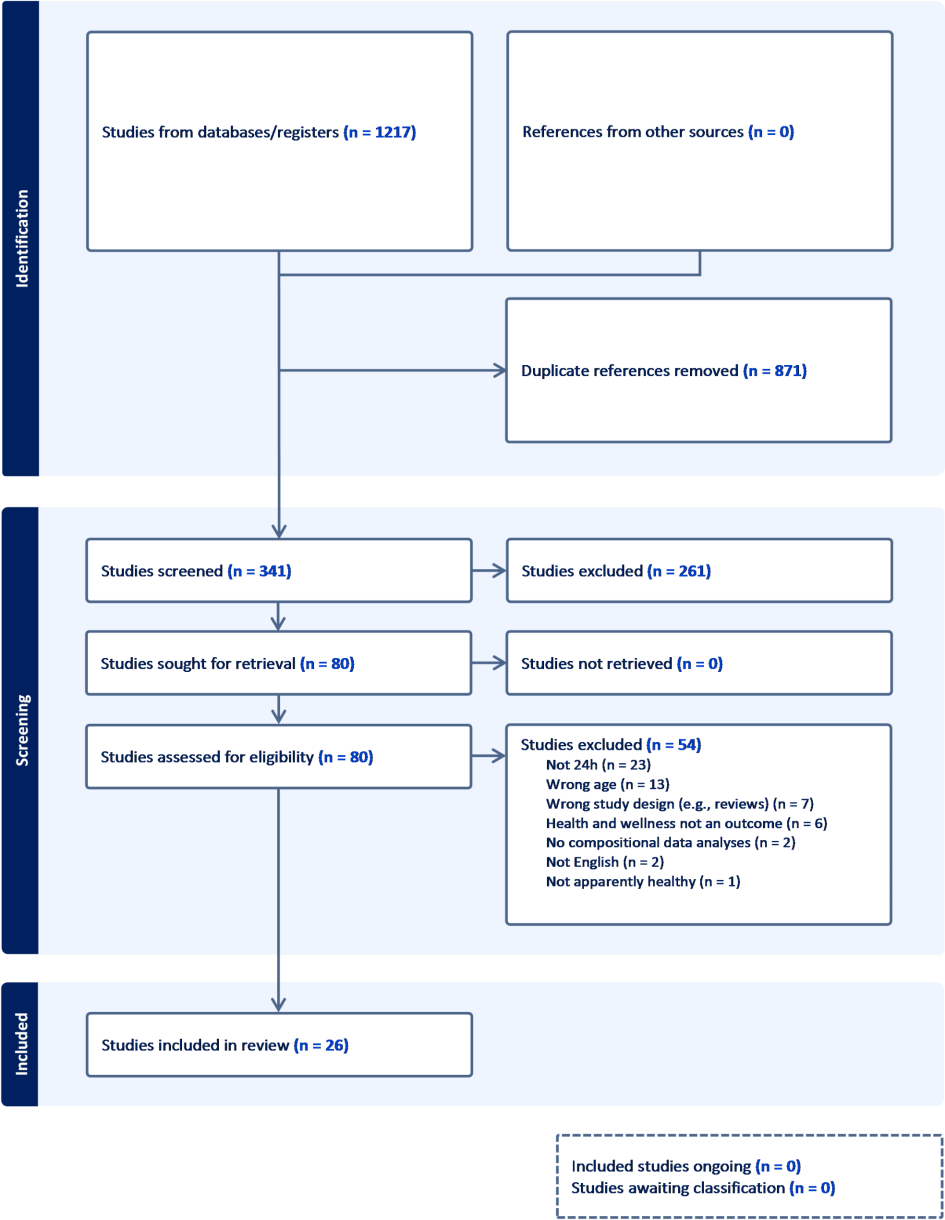
PRISMA flow diagram

In total, 1,217 studies were identified through database searches, after de-duplication and screening 54 studies were excluded at the full text stage (Additional File 3) leaving 26 included studies in this review (Fig. 1). Of the included studies, 16 reported compositional regression results [28, 33–47], 12 reported compositional substitution analysis results [28–30, 33, 34, 41, 45, 46, 48–51], three reported optimal movement behaviour composition results [16, 17, 52], and one study compared different clusters of movement behaviours [53]. Twenty-three studies presented cross-sectional analyses, while two studies presented both cross-sectional and prospective longitudinal analyses [35, 46], and one study presented prospective longitudinal analyses alone [50]. Sample sizes of the 26 included studies ranged from 88 to 5,828 (median = 387) participants, and the mean age ranged from 8.0 to 16.4 years (median = 11.7).

#### Study quality

When examining study quality by health and well-being outcome category, analysis type, and longitudinal/cross-sectional study design, only four groupings were classified as moderate study quality (range: 57–79.8%), while all others were classified as high study quality (Table 1). The Overall quality across study designs and analysis types was high quality, except for longitudinal regression, which was moderate quality (79.8%).

**Table 1:** Study quality summary by design and outcome category

Health and Well-Being Outcome Category	Analysis	Outcome Variables Assessed (*n*)	Studies (k)	Study Quality (%)
*Cross-sectional Designs*				
Overall	Goldilocks	24	3	88.2
	Regression	54	16	85.1
	Substitution	35	13	85.4
Adiposity Indicators	Goldilocks^1^	5	2	74.0
	Regression^2^	12	8	83.3
	Substitution^3^	13	8	85.7
Cardiometabolic Biomarkers	Goldilocks^4^	2	1	86.0
	Regression^5^	11	2	86.0
	Substitution^6^	6	1	86.0
Cognitive Indicators	Goldilocks^7^	3	1	86.0
	Regression^8^	5	2	86.0
Fitness	Goldilocks^9^	1	1	86.0
	Regression^10^	1	1	86.0
	Substitution^11^	6	3	88.3
Mental Well-Being and Illness Indicators	Goldilocks^12^	4	1	86.0
	Regression^13^	23	6	85.8
	Substitution^14^	9	3	81.1
Motor Skills	Regression^15^	1	1	100.0
	Substitution^16^	1	1	100.0
Musculoskeletal Growth	Goldilocks^17^	9	2	98.4
Other Health Behaviours	Regression^18^	1	1	57.0
*Longitudinal Designs*				
Overall	Regression	12	2	79.8
	Substitution	14	2	93.0
Cardiometabolic Biomarkers	Substitution^19^	7	1	100.0
Mental Well-Being and Illness Indicators	Regression^20^	12	2	79.8
	Substitution^21^	7	1	86.0

Study [outcome measure]

1: Dumuid, et al. [16] [Body fat %]; Lund Rasmussen, et al. [52] [BMI z-score, fat mass percent, fat mass index, visceral adipose tissue]

2: Carson, et al. [33] [BMI z-score, waist circumference]; Chen, et al. [34] [BMI z-score]; Dumuid, et al. [38] [BMI z-score]; Dumuid, et al. [37] [BMI z-score]; Gaba, et al. [41] [Fat mass %, fat mass index]; Haszard, et al. [28] [BMI z-score]; Matricciani, et al. [42] [BMI z-score]; Talarico and Janssen [45] [BMI z-score, waist circumference, fat mass index]

3: Carson, et al. [33] [BMI z-score, waist circumference]; Chen, et al. [34] [BMI z-score]; Domingues, et al. [30] [BMI z-score]; Dumuid, et al. [29] [Body fat %]; Fairclough, et al. [48] [BMI z-score, percentage waist circumference-to-height ratio]; Gaba, et al. [41] [Fat mass %, fat mass index]; Haszard, et al. [28] [BMI z-score]; Talarico and Janssen [45] [BMI z-score, waist circumference, fat mass index]

4: Dumuid, et al. [16] [Mean arterial blood pressure, inflammation (glycoprotein acetylation)]

5: Carson, et al. [33] [Systolic blood pressure, diastolic blood pressure, triglycerides, high density lipoprotein cholesterol, c-reactive protein, insulin]; Matricciani, et al. [42] [Metabolic syndrome severity score, systolic blood pressure, diastolic blood pressure, glycoprotein acetyls, apolipoprotein B/A1]

6: Carson, et al. [33] [Systolic blood pressure, diastolic blood pressure, triglycerides, high density lipoprotein cholesterol, c-reactive protein, insulin]

7: Dumuid, et al. [16] [Non-verbal IQ, receptive vocabulary, academic performance]

8: Fairclough, et al. [40] [Switching errors, spatial working memory errors, inhibition errors]; Watson, et al. [47] [Literacy, numeracy]

9: Dumuid, et al. [16] [Cardiorespiratory fitness]

10: Carson, et al. [33] [modified Canadian Aerobic Fitness Test]

11: Carson, et al. [33] [modified Canadian Aerobic Fitness Test]; Fairclough, et al. [48] [VO^2^ peak]; Zhang, et al. [51] [Physical fitness score, 50-metre run, long-distance running score, standing long jump]

12: Dumuid, et al. [16] [Life satisfaction, psychosocial health, depression, emotional problems]

13: Carson, et al. [33] [Strength and difficulties]; Chong, et al. [35] [Strength and difficulties internalising problems, externalizing problems, total difficulties, prosocial behaviour, psychological distress]; Dumuid, et al. [36] [Health-related quality of life]; Fairclough, et al. [39] [Strength and difficulties total difficulties, externalizing problems, internalizing problems]; Fairclough, et al. [40] [Self-esteem, depression, strength and difficulties total difficulties, internalizing problems, externalizing problems, prosocial behaviour]; Tan, et al. [46] [Health related quality of life, physical well-being, emotional well-being, self-esteem, relationship with family, relationship with friends, school functioning]

14: Carson, et al. [33] [Strength and difficulties]; Faria, et al. [49] [depression/anxiety Portuguese 12-item General Health Questionnaire]; Tan, et al. [46] [Health related quality of life, physical well-being, emotional well-being, self-esteem, relationship with family, relationship with friends, school functioning]

15: Smith, et al. [44] [Fundamental movement skill competency]

16: Smith, et al. [44] [Fundamental movement skill competency]

17: Dumuid, et al. [17] [Peripheral quantitative computed tomography scan ankle (4% site) cross-sectional area, ankle (4% site) trabecular density, shin (66% site) cortical density, shin (66% site) endosteal circumference, shin (66% site) periosteal circumference, polar moment of inertia, polar stress-strain index, overall bone zone]; Dumuid, et al. [16] [Bone strength]

18: Ren, et al. [43] [Smartphone addiction prevalence]

19: Segura-Jimenez, et al. [50] [C3 complement factor (inflammatory marker), C4 complement factor (inflammatory marker), leptin, tumor necrosis factor, C-reactive protein, adiponectin, interleukin-6]

20: Chong, et al. [35] [Strengths and difficulties internalising problems, externalising problems, total difficulties, prosocial behaviour, psychological distress]; Tan, et al. [46] [Health related quality of life, physical well-being, emotional well-being, self-esteem, relationship with family, relationship with friends, school functioning]

21: Tan, et al. [46] [Health related quality of life, physical well-being, emotional well-being, self-esteem, relationship with family, relationship with friends, school functioning]

#### Descriptive movement behaviours

**Fig. 2:**
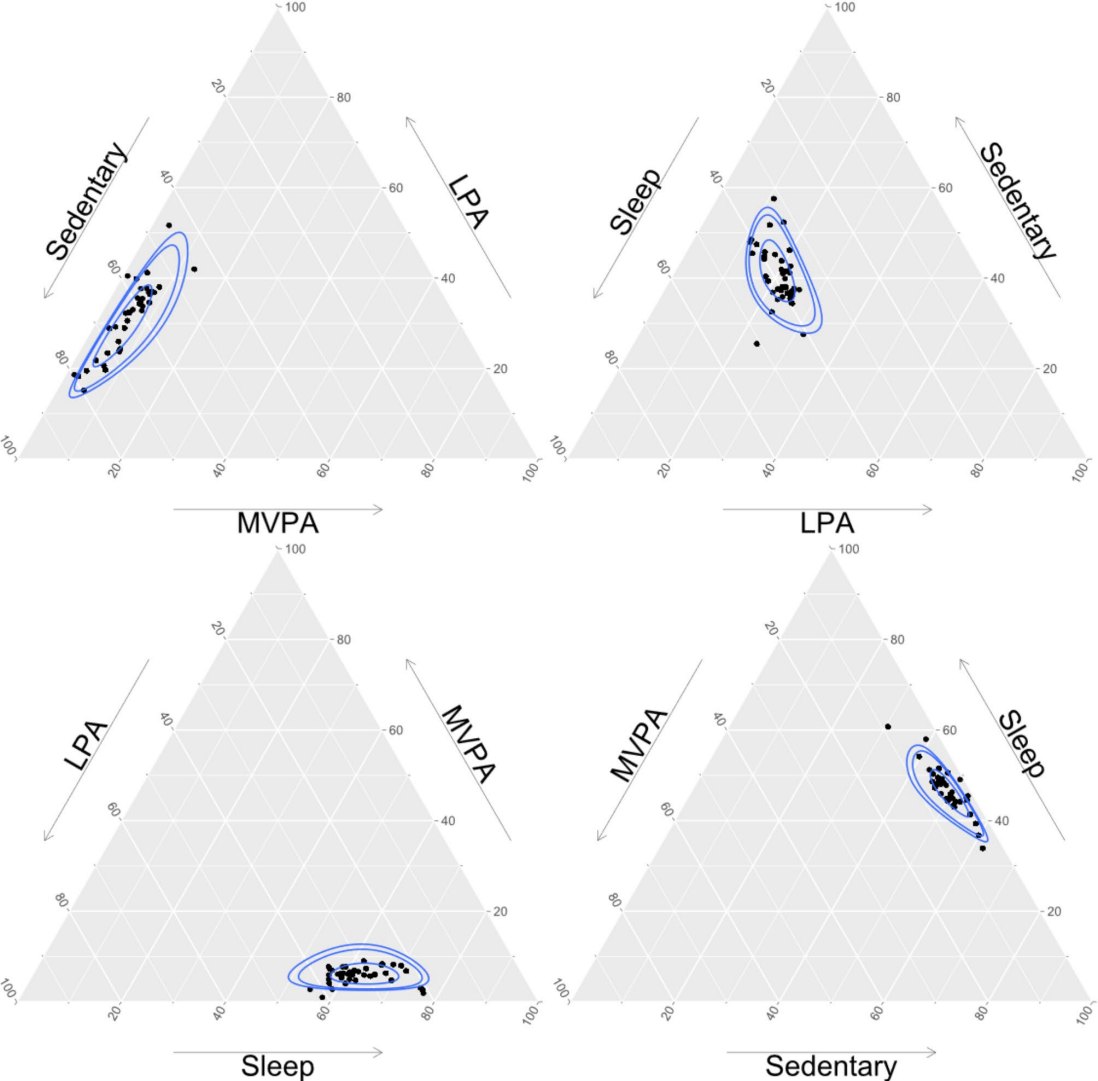
Ternary plots of the distribution of 4 main movement behaviours. Individual dots represent unique mean movement behaviour compositions (*n* = 39 samples or sub-samples) reported in the 22 studies reporting sleep, sedentary behaviour, light physical activity, and moderate to vigorous physical activity. Rings surrounding dots represent 50th, 90th, and 95th percentile confidence intervals. LPA = light physical activity, MVPA = moderate to vigorous physical activity

All 26 studies examined total sleep, with no sub-components; 25 of 26 studies reported total sedentary time with the remaining study breaking sedentary time down into in- and out-of-school components (See Additional File 4 for full study details). In terms of physical activity constructs, total physical activity was used in one study. In 22 studies, physical activity was separated into LPA and MVPA components, and an additional 2 studies examined LPA, moderate physical activity (MPA), and vigorous physical activity (VPA) components. Finally, one study examined physical activity by including in- and out-of-school LPA, MPA, and VPA. Seventeen studies reported results across the entire sample, while the other 9 studies reported only stratified results across a total of *k*_strata_=34 subgroups (i.e., age groups k _strata_=4, clusters *k*_strata_=8, countries *k*_strata_=12, girls/boys *k*_strata_=4, study cohorts *k*_strata_=2, timepoints *k*_strata_=4), resulting in 51 samples contributing unique summary data points for synthesis. Across these 51 included samples, sleep was the most frequently reported movement behaviour from the 26 studies with 51 unique central tendency values reported, followed by sedentary behaviour with 49, LPA with 48, and MVPA with 39. The compositional mean and standard deviation, weighted by sample/sub-sample size, of movement behaviours are presented in Table 2, while ternary plots of the distribution of sleep, sedentary behaviour, LPA, and MVPA are presented in Fig. 2. Notably, only nine studies reported movement behaviour dispersion metrics, including standard deviations [16, 28, 30, 34, 35, 46, 52] or 95% confidence intervals [17, 50].

**Table 2:** Weighted central tendency of most commonly reported movement behaviours

Movement Behaviours*	Weighted Mean (SD)	Accelerometer Studies/Total Studies	Frequency of unique values
Sleep	545.0 (26.3)	22/26	51
Sedentary Behaviour	535.0 (54.7)	23/25	49
LPA	308.0 (45.7)	23/24	48
MVPA	49.8 (16.8)	21/22	39
MPA	42.6 (10.0)	2/2	9
VPA	16.7 (6.1)	2/2	9

*Movement behaviours represent the total durations accumulated in a 24-hour day, unless otherwise indicated (e.g., Out-of-school MVPA). All compositional mean and standard deviation values are in minutes/day, and weighted by individual study sample sizes.

SD = standard deviation, LPA = light physical activity, MVPA = moderate to vigorous physical activity, MPA = moderate physical activity, VPA = vigorous physical activity

### Regression analyses

**Fig. 3:**
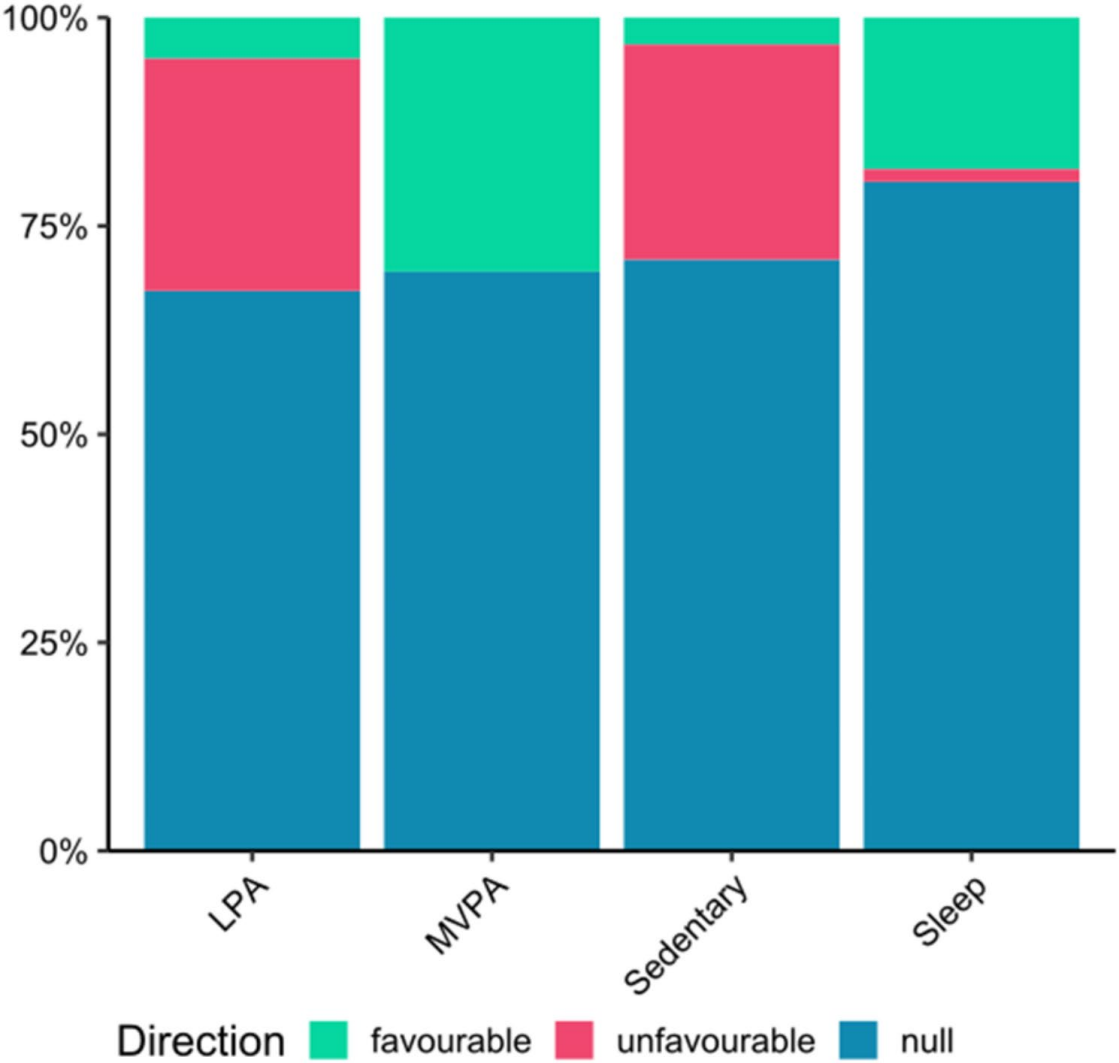
Overall direction of associations in compositional regression analyses of the four main movement behaviours: light physical activity (LPA), moderate to vigorous physical activity (MVPA), sedentary behaviour (Sedentary), and sleep

Results were generally null for health and well-being when examining regression analyses for sleep, sedentary behaviour, LPA, and MVPA, though some favourable trends were observed for MVPA and unfavourable trends for LPA and sedentary behaviour within individual outcome categories (Table 3). Specifically, all associations with adiposity indicators (10/10) and the majority with cardiometabolic biomarkers (9/11) were favourable for MVPA. Conversely, LPA was unfavourable for adiposity indicators (8/10) and cognitive indicators (5/7), while sedentary behaviour was unfavourable for adiposity indicators (7/10) alone. Trends in null findings were observed for many movement behaviours and health and well-being categories, and for all movement behaviours in the Overall category that combined all examined associations (Table 3; Fig. 3). The direction of all associations, including the less commonly examined movement behaviours (e.g., VPA, in-school MVPA) can be found in Additional File 5.

**Table 3:** Direction of associations for compositional regression analyses

Health and Well-Being Categories	Exposure	F	U	*N*	Total
Overall	LPA	3	18	**46**	67
	MVPA	23	0	**42**	65
	Sedentary	2	19	**47**	68
	Sleep	14	1	**57**	72
Adiposity Indicators	LPA	1	**8**	1	10
	MVPA	**10**	0	0	10
	Sedentary	0	**7**	4	11
	Sleep	6	1	**8**	15
Cardiometabolic Biomarkers	LPA	0	1	**10**	11
	MVPA	**9**	0	2	11
	Sedentary	0	2	**9**	11
	Sleep	2	0	**9**	11
Cognitive Indicators	LPA	1	**5**	1	7
	MVPA	0	0	**7**	7
	Sedentary	2	0	**5**	7
	Sleep	0	0	**7**	7
Fitness	LPA	0	0	1	1
	MVPA	1	0	0	1
	Sedentary	0	1	0	1
	Sleep	0	0	1	1
Mental Well-Being and Illness Indicators*	LPA	1	3	**32**	36
	MVPA	2	0	**32**	34
	Sedentary	0	8	**28**	36
	Sleep	5	0	**31**	36
Motor skills	LPA	0	0	1	1
	MVPA	0	0	1	1
	Sedentary	0	0	1	1
	Sleep	0	0	1	1
Other Health Behaviours	LPA	0	1	0	1
	MVPA	1	0	0	1
	Sedentary	0	1	0	1
	Sleep	1	0	0	1

F = Favourable, U = Unfavourable, N = Null, LPA = light physical activity, MVPA = moderate to vigorous physical activity, * = 12/36 associations are longitudinal from two studies, with all being null except for 3/12 unfavourable associations for sedentary behaviour. Bolded values indicate more than 50% of associations are in that direction, when more than one association is examined

### Substitutions

Substitutions involving MVPA were significant in meta-analyses for overall health outcome changes (Table 4 and Additional File 6). Specifically, substitutions replacing sedentary behaviour, sleep, or LPA for 10 min more MVPA were associated with a 0.1 standardized mean difference (SMD) increase in overall health and well-being (Table 4). Replacing MVPA with 10 min of sleep, sedentary behaviour, and LPA resulted in a larger decrease in overall health and well-being (-0.2 SMD) compared to adding MVPA (0.1 SMD).

**Table 4:** Meta-analyses of movement behaviour substitutions for overall health and well-being

Study Design	Comparison	SMD (95% CI)*	# Studies
***Cross-Sectional***	More LPA, Less MVPA	**-0.17 (-0.07**,** -0.27)**	9
	More LPA, Less Sedentary	-0.03 (0.02, -0.09)	10
	More LPA, Less Sleep	-0.04 (0.00, -0.09)	9
	More MVPA, Less LPA	**0.11 (0.17**,** 0.05)**	10
	More MVPA, Less Sedentary	**0.11 (0.18**,** 0.04)**	9
	More MVPA, Less Sleep	**0.07 (0.13**,** 0.01)**	9
	More Sedentary, Less LPA	0.01 (0.07, -0.05)	10
	More Sedentary, Less MVPA	**-0.16 (-0.07**,** -0.26)**	9
	More Sedentary, Less Sleep	**-0.04 (-0.001**,** -0.07)**	9
	More Sleep, Less LPA	0.04 (0.09, -0.01)	9
	More Sleep, Less MVPA	**-0.16 (-0.03**,** -0.30)**	9
	More Sleep, Less Sedentary	0.03 (0.07, -0.00)	9
***Longitudinal***	More LPA, Less MVPA	0.00 (0.01, -0.00)	2
	More LPA, Less Sedentary	0.01 (0.02, -0.01)	2
	More LPA, Less Sleep	**-0.02 (-0.00**,** -0.04)**	2
	More MVPA, Less LPA	0.01 (0.03, -0.01)	2
	More MVPA, Less Sedentary	**0.01 (0.01**,** 0.01)**	2
	More MVPA, Less Sleep	**-0.01 (-0.01**,** -0.01)**	2
	More Sedentary, Less LPA	0.00 (0.04, -0.03)	2
	More Sedentary, Less MVPA	0.00 (0.02, -0.01)	2
	More Sedentary, Less Sleep	**-0.03 (-0.02**,** -0.04)**	2
	More Sleep, Less LPA	0.04 (0.08, -0.01)	2
	More Sleep, Less MVPA	0.02 (0.04, -0.00)	2
	More Sleep, Less Sedentary	**0.04 (0.07**,** 0.01)**	2

SMD = Standard mean difference in random effects models, LPA = light physical activity, MVPA = moderate to vigorous physical activity. Bolded values indicate statistical significance according to 95% confidence intervals

### Optimal movement behaviour distributions

Of the three studies examining the Goldilocks Day or the optimal distribution of movement behaviours, one study examined adiposity indicators, another study examined bone health, and a third study examined a range of health outcomes that included both bone health and adiposity indicators. For bone health, the two studies used data from the Child Health CheckPoint study, thus the main bone health outcome data were the same (Polar Stress-Strain Index resistance) in both analyses. However, Dumuid, et al. [16] and Lund Rasmussen, et al. [52] both examined the ideal distribution of movement behaviours for adiposity indicators using separate datasets, so the weighted mean and range of movement behaviours for adiposity indicators were calculated in Table 5. Finally, a narrative summary of the studies examining the ideal distribution of movement behaviours for all health could simply summarize Dumuid, et al. [16] where an ideal day of movement behaviours across mental, cognitive/academic, and physical health consisted of 10.4 h/day of sleep, 9.7 h/day of sedentary time, 2.4 h/day of LPA, and 1.5 h/day of MVPA.

**Table 5:** Weighted mean ideal day of movement behaviours for optimal adiposity indicators

Study	Group	Sleep^a^	Sedentary^a^	LPA^a^	MVPA^a^
Dumuid, et al. [16]^**b**^	—	10.7	9.3	1.9	2.1
Lund Rasmussen, et al. [52]	Children	8.5 (6.4, 10.1)	10.8 (8.3, 14)	3.9 (2.1, 6.6)	0.8 (0.1, 2.5)
Lund Rasmussen, et al. [52]	Adolescents	7.5 (5.7, 9.8)	12.4 (9.5, 15.5)	3.6 (1.7, 6.6)	0.5 (0.1, 1.4)
**Weighted Mean** **(min**,** max)**		**9.8** **(5.7**,** 10.1)**	**10.1** **(8.3**,** 15.5)**	**2.5** **(1.7**,** 6.6)**	**1.6** **(0.1**,** 2.5)**

^a^Movement behaviours are expressed as mean (range) hours/day, with weighted means calculated by weighting for sample/sub-sample sizes.

^b^Sample consisted of children (11–12 years of age)

Sedentary = sedentary behaviour, LPA = light physical activity, MVPA = moderate to vigorous physical activity

## Discussion

This systematic review presents the first meta-analysis of CoDA movement behaviour substitutions across several health and well-being domains in school-aged children and adolescents. Further, a comprehensive summary of health and well-being for this age group within CoDA regression and Goldilocks Day results was synthesized. Generally, this review reinforces previous reviews identifying the importance of MVPA, which is a troubling finding considering the weighted mean estimate of MVPA across the included studies is below the recommended 60 min/day. In relation to the rest of the compositional movement behaviours, MVPA was particularly important for adiposity indicators and biomarkers in CoDA regression models. Substitution models also demonstrated the importance of MVPA in cross-sectional analyses, though effect sizes were small. Lastly, while there were limited results presenting the Goldilocks Day, the weighted mean optimal day for adiposity indicators across 2 studies (three groups) was calculated as 9.8 h/day of sleep, 10.1 h/day of sedentary behaviour, 2.5 h/day of LPA, and 1.6 h/day of MVPA.

Within the regression, substitution, and Goldilocks results, the benefits of MVPA were the most apparent, as reflected in previous reviews [1, 19, 21]. Bourke, et al. [21] meta-analyzed the effects of one-for-all substitutions on adiposity indicators and found that increasing MVPA was the most beneficial movement behaviour substitution. Despite differences between Bourke, et al. [21] and the current review (e.g., the current review examined all health and well-being outcomes, predominantly one-for-one substitutions, excluded early years children) the benefits of MVPA are a mutually resonating message. While the argument could be made that MVPA should be prioritized in guideline recommendations, since, compared to other movement behaviours, MVPA consistently has the lowest adherence and the strongest evidence for health and well-being benefits. However, the current review and Bourke, et al. [21] found evidence supporting the importance of the whole 24-hour day. Specifically, Bourke, et al. [21] found decreasing sedentary time and LPA, as well as increasing sleep was favourable for adiposity indicators. Likewise, 10-minute substitutions increasing sedentary time at the expense of sleep were unfavourable for overall health and well-being in the current review. Further, when examining the direction of regression coefficients in the adiposity domain, MVPA was favourable, while sedentary time and LPA were unfavourable. Thus, both reviews ultimately point out the strong benefits of MVPA, and the importance of the 24-hour movement behaviour approach of accumulating more physical activity and sleep, while decreasing sedentary time. Further examining the benefits of MVPA presented in this review, cross-sectional substitutions replacing 10 min of sleep, sedentary behaviour, or LPA with MVPA were associated with a 0.1 standardized mean difference (SMD) increase in overall health and well-being. For example, 0.1 SMDs were calculated for Chen et al., [31] based on a -0.1 BMI z-score change when middle school students exchanged 10 min of sleep or sedentary behaviour for MVPA. Whereas the reciprocal substitutions that replace 10 min of MVPA for one of the other three movement behaviours were associated with a -0.2 SMD decrease in overall health and well-being. Beyond the need to promote MVPA, this speaks to the urgency of protecting the limited MVPA school-age children currently accumulate. While the importance of MVPA was also demonstrated in longitudinal substitutions (i.e., 0.01 SMD overall health and well-being when substituting 10 min of sedentary for MVPA), the benefits of sleep were more apparent (e.g., -0.01 SMD overall health and well-being when substituting 10 min of sleep for MVPA). However, the effect sizes were very small and the results relied on only two studies. Future meta-analyses should re-examine longitudinal substitutions to test our preliminary results that the benefits of sleep may surpass MVPA over time. One issue with relying on meta-analyses of substitution results is that the substitutions are inherently dependent on the baseline or reference composition of movement behaviours. For instance, the effect of adding 10 min of MVPA at the expense of sedentary behaviour depends on the initial composition of movement behaviours (e.g., adding 10 min is likely more beneficial for someone with 15 total minutes/day of MVPA compared to someone with 120 min/day of MVPA). Individual studies often set the substitution reference point to the geometric mean of the sample. However, as demonstrated in this review, these reference points vary between studies (often by more than the 10 min typically used for substitution effects). In a self-contained example of this, Fairclough, et al. [48] noted that “*results of compositional isotemporal substitution…will differ according to the baseline activity composition*” and indeed reported different 15-minute substitution effects based on three different reference compositions (corresponding to the movement behaviours of underweight, normal weight, and overweight/obese children in the sample). Granted, it could be assumed that for the average school-aged child (Table 2: 9.1 h/sleep, 8.9 h/day of sedentary behaviour, 5.1 h/day LPA, and 0.8 h/day of MVPA) these substitutions would have the desired effects, but it still lacks the precision needed for advancing children’s health and well-being [54].

Summarizing Goldilocks Day results could advance the precision needed to improve children’s health and well-being by allowing researchers to move closer to understanding the optimal balance of movement behaviours. However, the measurement of movement behaviours is still an important consideration. For instance, when comparing the ideal day for optimal polar stress-strain index in the two included studies using the Child Health CheckPoint dataset, Dumuid, et al. [16] reported 0.6 h/day less sleep, 2.0 h/day less sedentary behaviour, 1.3 h/day more LPA, and 1.3 h/day more MVPA, compared to Dumuid, et al. [17]. Comparing the two studies, the baseline movement behaviours differ while the protocols and data treatment rules are nearly the same, except Dumuid, et al. [16] weighted the accelerometer data to represent weekend and weekday data (i.e., [mean hours/weekdays * 5 + mean hours/weekend day * 2] / 7) while Dumuid, et al. [17] did not. It is alarming that a seemingly innocuous decision, with no clear consensus of “correctness” one way or another could swing the guidance for an optimal day of MVPA by over an hour. Understanding the optimal distribution of movement behaviours is paramount for generating precise recommendations that enhance children’s health and well-being, and we recommend researchers continue adding to this literature using the techniques discussed in the included optimal movement behaviour literature [16, 17, 52]. As well, more efforts are needed to reach consensus on universally accepted accelerometer data collection and processing decisions [55], to ensure confidence in future optimal distribution recommendations.

### Future directions for movement behaviour CoDA researchers

In addition to establishing consensus on accelerometer data collection and processing, it is crucial to develop standardized guidelines for reporting CoDA results [56]. We agree with Brown, et al. [56] that creating an observational CoDA reporting checklist through DELPHI consensus would significantly enhance the feasibility and confidence of future meta-analyses. Based on our current efforts in CoDA meta-analyses, we propose the following additional considerations:

1. Only 9/26 studies reported dispersion metrics for movement behaviours. Without this information, there will be a vague level of confidence for the movement behaviours of school-aged children. Further, understanding why some substitutions are beneficial, while equal substitutions are null or unfavourable in different studies could lie in better understanding baseline movement behaviours. For instance, if a sample generally has sufficient sleep then it would be intuitive that adding sleep may not be beneficial on average, whereas adding sleep to a sample with insufficient sleep could prove very beneficial.

2. When models are built to predict health and well-being outcomes, all CoDA model parameter estimates should be reported, rather than just the estimate for the first ilr pivot. This will allow future researchers to better replicate results, meta-analyze the predictive formulas, or generate predicted results that meet specific needs (e.g., estimate substitution effects with different reference compositions). An inherent limitation of our substitution analysis was having to standardize exposures to a 10-minute increment using a linear transformation despite knowing that CoDA results frequently demonstrate a curvilinear association between the composition exposure variables and the regressand. Imposing linear transformations on potentially non-linear associations may distort the true results. As well, some would argue that absolute units (e.g., 10 min) are valuable for interpretation and messaging, but proportional units (e.g., 10% of behaviour) better represent the unequal distribution of movement behaviours in a composition. For instance, a 10 min substitution could seem strong for MVPA and weaker for sleep, since 10 min could be 20% of MVPA and 2% of sleep. Having access to full parameter estimates for the model would have avoided this limitation by allowing us to run our own substitution analyses using time increments of our choosing and select a common starting point for all substitutions (e.g. relative to an aggregated sample mean across all studies). Brown, et al. [56] have recommended reporting standardized regression coefficients (i.e. beta coefficients). However, this may not always be an appropriate measure of effect, especially when the units of either the regressor or the regressand have meaningful units or the distribution of either variable is non-normal [57]. Rather, we would encourage authors to clearly state whether they are reporting standardized, unstandardized, or half-standardized (i.e. where only the regressand is converted to a Z-score) effects and provide rationale for their choice and sufficient information to back-transform standardized effects into their non-standardized counterparts (i.e. mean and standard deviation of the regressors and regressand). Further, only two studies reported testing quadratic, or squared, terms to improve model fit [16, 17]. Testing squared terms could help improve the precision for CoDA models, but at a minimum reporting when terms are linear should be standard to improve future replication studies and meta-analyses.

3. Particularly for accelerometer studies that report the geometric means of movement behaviours, not reporting non-wear time or arithmetic means of movement behaviours could provide misleading results [28]. For two of the included studies, only arithmetic means of movement behaviours were reported [28, 46]. Presenting only the normalized geometric mean of the included variables can obfuscate how much time use typically went unmeasured amongst participants due to device non-wear or non-report. Normalizing the geometric mean (e.g., to 1,440 min) acts as a type of person mean imputation where the composition of the unmeasured time is assumed to exist in the same ratios as what was measured. For instance, a school-aged child with 7.00 h/day of sleep, 7.00 h/day of sedentary behaviour, 2.25 h/day of LPA, and 0.75 h/day of MVPA when scaled to a 24-hour composition would result in 9.9 h/day of sleep and 1.1 h/day of MVPA—or go from not meeting 24-hour movement guidelines for sleep and MVPA, to meeting both guidelines. Haszard, et al. [28] suggest this scaling should not include sleep, since non-wear time is typically accumulated during waking hours, not during sleep. Applying this technique to the hypothetical school-aged children would retain the 7 h of insufficient sleep, but MVPA would still exceed guideline recommendations with 1.3 h/day. Regardless, identifying an optimal day of movement behaviours based on best guesses as to where to hide the elephant in the room that is non-wear time is problematic. Numerous techniques have been developed to impute replacements for non-wear time when epoch-by-epoch accelerometer data is available [58, 59] and are likely the best option for mitigating the potential impact of non-wear time on the conclusions drawn from CoDA of movement behaviours. When this level of data granularity is not available, CoDA for data with a meaningful total [60, 61] may be a viable option to account for incomplete days. Regardless, even if authors choose to use person mean imputation through geometric mean normalization, reporting arithmetic means is an easy solution to address missing data, as the sum total of behaviours will illustrate the total wear time and allow the reader to intuitively see the difference with the geometric mean.

### Strengths and limitations

The primary strength of this review is presenting the first meta-analyses of CoDA movement behaviour studies across several health and well-being outcome domains, while simultaneously providing a comprehensive synthesis of the existing literature on compositional movement behaviours and their associations with health and well-being in school-aged children. However, several limitations arose while achieving the study objectives. One challenge was the heterogeneity of outcomes and measures across the included studies. For instance, the overall health category combined results across all health and well-being categories (e.g., mental health and well-being, adiposity indicators). While indicating the benefits for overall health and well-being is a concise and powerful public health message, it may lose some nuances important for specific domains of health and well-being. Additionally, in this review we decided to use the combined overall health and well-being category for meta-analysis to buffer for the low number of studies in individual health and well-being outcome categories. Similar heterogeneity arguments could be made for individual outcome categories, such as adiposity indicators where it is presumed that results for body mass index z-scores and body fat percentages have equal predictive validity in discerning health risk for school-aged children. Further, when grouping longitudinal studies together some variance existed between studies, such as reporting on a single movement behaviour timepoint in relation to changes in an outcome [35], versus reporting on changes in movement behaviours across two timepoints in relation to changes in an outcome [50]. Ideally, as the literature on CoDA of movement behaviours continues to progress and accumulate, future meta-analyses can learn from this review and perform more targeted analyses. This is a particularly salient point considering the a priori research questions could not be addressed with the extracted data, and many questions remain regarding: the optimal distribution, patterns, and relevant covariates associated with movement behaviours and health and well-being in children and adolescents. Additionally, this review may have included further studies if additional steps were taken in the search strategy (e.g., backward and forward citation searches, targeted search of relevant journals such as the Journal of Activity, Sedentary and Sleep Behaviors).

## Conclusion

This systematic review and meta-analysis quantitatively synthesized CoDA of movement behaviours for school-aged children’s health and well-being across the three main analysis categories of (1) direction of associations for linear regression analyses, (2) standardized mean differences when substituting movement behaviours, and (3) determining the weighted mean of the optimal movement behaviour distributions (or Goldilocks days). Results should be interpreted with caution due to the heterogeneity of studies and outcome categories used within meta-analyses. However, these results reinforce previous findings demonstrating the need to promote MVPA, and perhaps more importantly preserve the limited MVPA school-age children currently accumulate. Further, some evidence supported the 24-hour movement behaviour messaging of increasing sleep and decreasing sedentary behaviour. As research on CoDA of movement behaviours progresses and accumulates further findings, the current review can serve as a stepping stone toward producing meta-analyses to inform precision health guidance needed for optimizing children’s health and well-being.

## Electronic supplementary material

Below is the link to the electronic supplementary material.

Supplementary Material 1

Supplementary Material 2

Supplementary Material 3

Supplementary Material 4

Supplementary Material 5

Supplementary Material 6

## Data Availability

No datasets were generated or analysed during the current study.
