# Supplementary material for: A systematic review and meta-analysis of the first decade of compositional data analyses of 24-hour movement behaviours, health, and well-being in school-aged children
Source: J Act Sedentary Sleep Behav. 2025 Mar 27;4:4. doi: 10.1186/s44167-025-00076-w (PMC11948812; doi:10.1186/s44167-025-00076-w)
Supplement: Supplementary file 2 — Supplementary Material 2 [file 44167_2025_76_MOESM2_ESM.pdf]

Study	SMD	95%−CI	Weight (common)	Weight (random)
Carson, et al. (33)	0.00	[0.00; 0.00]	96.3%	10.0%
Domingues, et al. (30)	−0.01	[−0.01; 0.00]	0.1%	10.0%
Dumuid, et al. (29)	−0.01	[−0.01; −0.00]	1.8%	10.0%
Fairclough, et al. (48)	−0.02	[−0.03; −0.02]	0.1%	10.0%
Haszard, et al. (28)	−0.19	[−0.19; −0.19]	0.8%	10.0%
Smith, et al. (44)	0.09	[0.08; 0.09]	0.1%	10.0%
Zhang, et al. (51)	−0.04	[−0.05; −0.04]	0.2%	10.0%
Faria, et al. (49)	−0.18	[−0.18; −0.17]	0.1%	10.0%
Chen, et al. (34)	0.03	[0.02; 0.04]	0.1%	10.0%
Tan, et al. (46)	0.02	[0.01; 0.02]	0.5%	10.0%
**Common effect model**	**0.00**	**[0.00; 0.00]**	**100.0%**	**.**
**Random effects model**	**−0.03**	**[−0.09; 0.02]**	**.**	**100.0%**

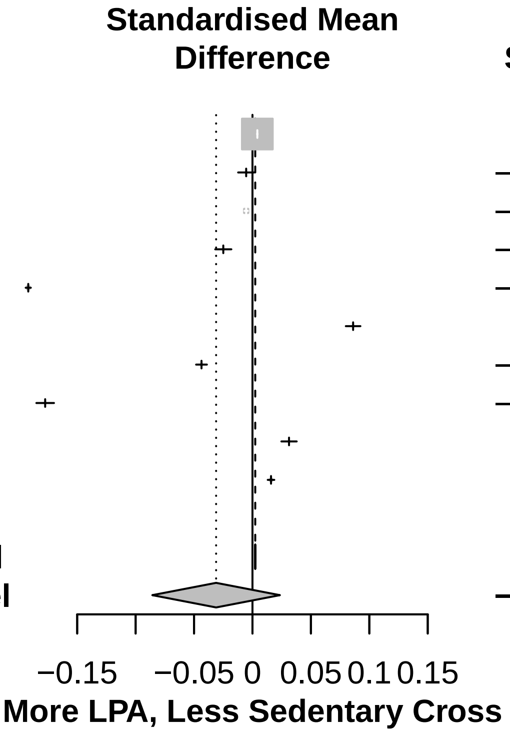

Heterogeneity: I^2 = 100%, p = 0

Study	SMD	95%−CI	Weight (common)	Weight (random)
Carson, et al. (33)	0.01	[0.01; 0.01]	96.4%	11.1%
Domingues, et al. (30)	−0.02	[−0.02; −0.01]	0.1%	11.1%
Dumuid, et al. (29)	0.07	[0.07; 0.07]	1.8%	11.1%
Fairclough, et al. (48)	0.24	[0.24; 0.25]	0.1%	11.1%
Haszard, et al. (28)	0.24	[0.24; 0.24]	0.8%	11.1%
Smith, et al. (44)	0.19	[0.19; 0.20]	0.1%	11.1%
Zhang, et al. (51)	0.05	[0.05; 0.06]	0.2%	11.1%
Chen, et al. (34)	0.17	[0.16; 0.17]	0.1%	11.1%
Tan, et al. (46)	0.02	[0.02; 0.02]	0.5%	11.1%
**Common effect model**	**0.01**	**[0.01; 0.01]**	**100.0%**	**.**
**Random effects model**	**0.11**	**[0.04; 0.18]**	**.**	**100.0%**

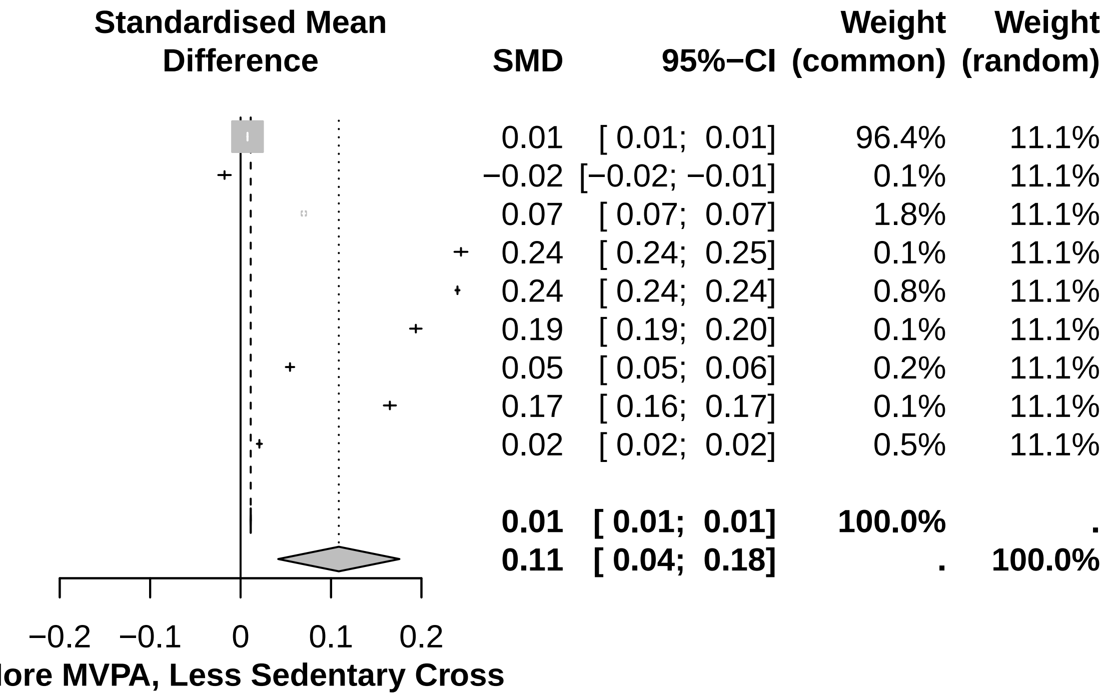

Heterogeneity: $I^2 = 100\%$, $p = 0$

Study	SMD	95%−CI	Weight (common)	Weight (random)
Carson, et al. (33)	0.01	[0.01; 0.01]	96.4%	11.1%
Domingues, et al. (30)	−0.00	[−0.01; 0.00]	0.1%	11.1%
Dumuid, et al. (29)	0.03	[0.03; 0.03]	1.8%	11.1%
Fairclough, et al. (48)	−0.01	[−0.02; −0.01]	0.1%	11.1%
Haszard, et al. (28)	0.11	[0.11; 0.11]	0.8%	11.1%
Smith, et al. (44)	0.12	[0.12; 0.13]	0.1%	11.1%
Zhang, et al. (51)	0.06	[0.06; 0.07]	0.2%	11.1%
Chen, et al. (34)	−0.00	[−0.01; 0.00]	0.1%	11.1%
Tan, et al. (46)	−0.01	[−0.01; −0.00]	0.5%	11.1%
**Common effect model**	**0.01**	**[0.01; 0.01]**	**100.0%**	**.**
**Random effects model**	**0.03**	**[−0.00; 0.07]**	**.**	**100.0%**

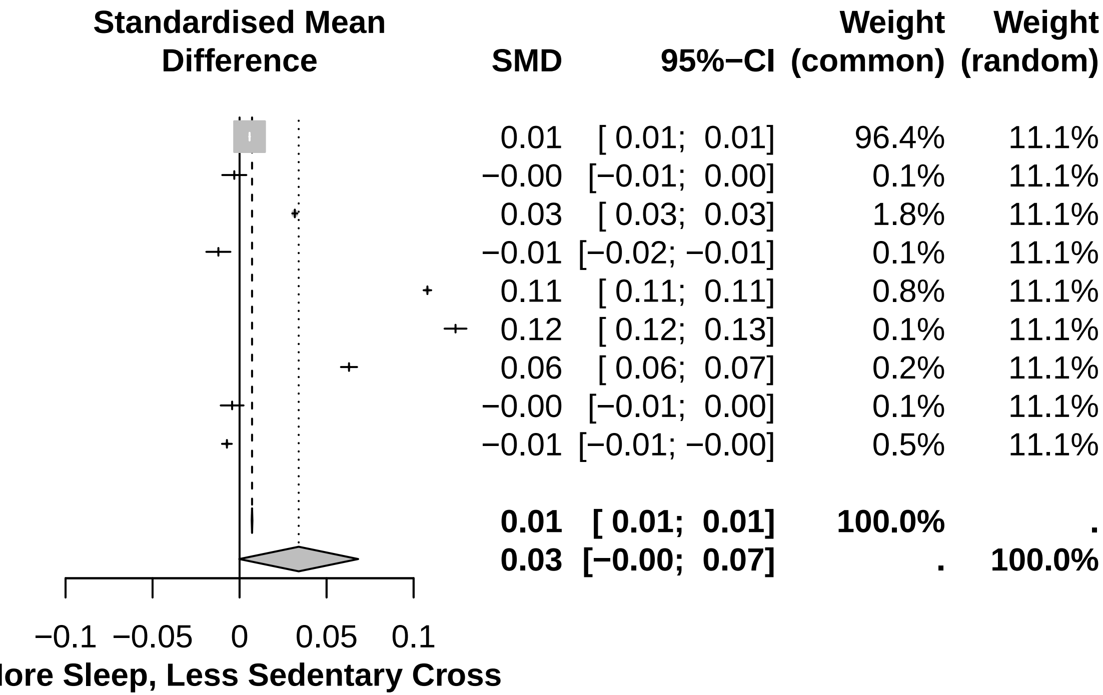

Heterogeneity: $I^2 = 100\%$, $p = 0$

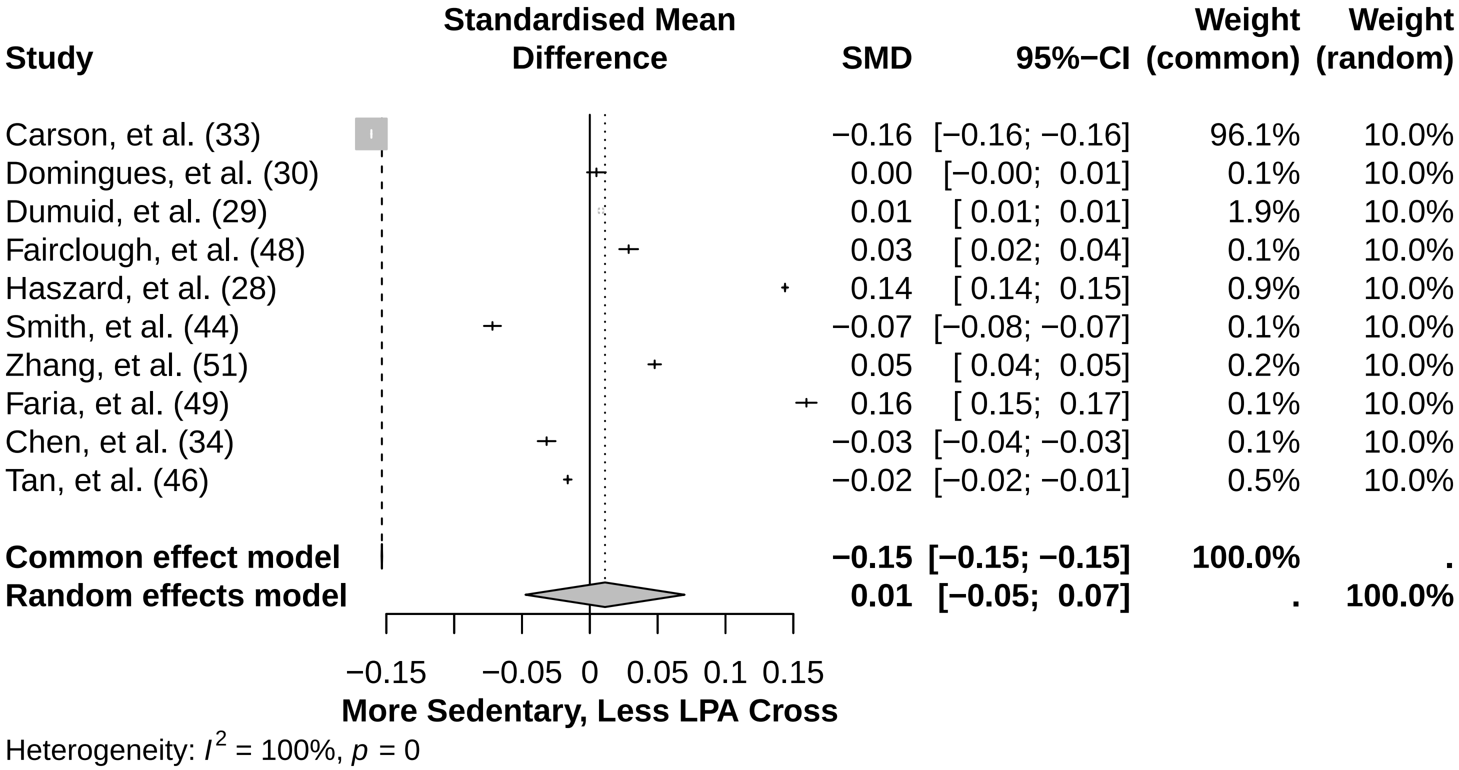
Study
Standardised Mean Difference
SMD
95%−CI
Weight (common)
Weight (random)
Carson, et al. (33) −0.16 [−0.16; −0.16] 96.1% 10.0%
Domingues, et al. (30) 0.00 [−0.00; 0.01] 0.1% 10.0%
Dumuid, et al. (29) 0.01 [0.01; 0.01] 1.9% 10.0%
Fairclough, et al. (48) 0.03 [0.02; 0.04] 0.1% 10.0%
Haszard, et al. (28) 0.14 [0.14; 0.15] 0.9% 10.0%
Smith, et al. (44) −0.07 [−0.08; −0.07] 0.1% 10.0%
Zhang, et al. (51) 0.05 [0.04; 0.05] 0.2% 10.0%
Faria, et al. (49) 0.16 [0.15; 0.17] 0.1% 10.0%
Chen, et al. (34) −0.03 [−0.04; −0.03] 0.1% 10.0%
Tan, et al. (46) −0.02 [−0.02; −0.01] 0.5% 10.0%
Common effect model −0.15 [−0.15; −0.15] 100.0% .
Random effects model 0.01 [−0.05; 0.07] . 100.0%
−0.15 −0.05 0 0.05 0.1 0.15
More Sedentary, Less LPA Cross
Heterogeneity: I2 = 100%, p = 0

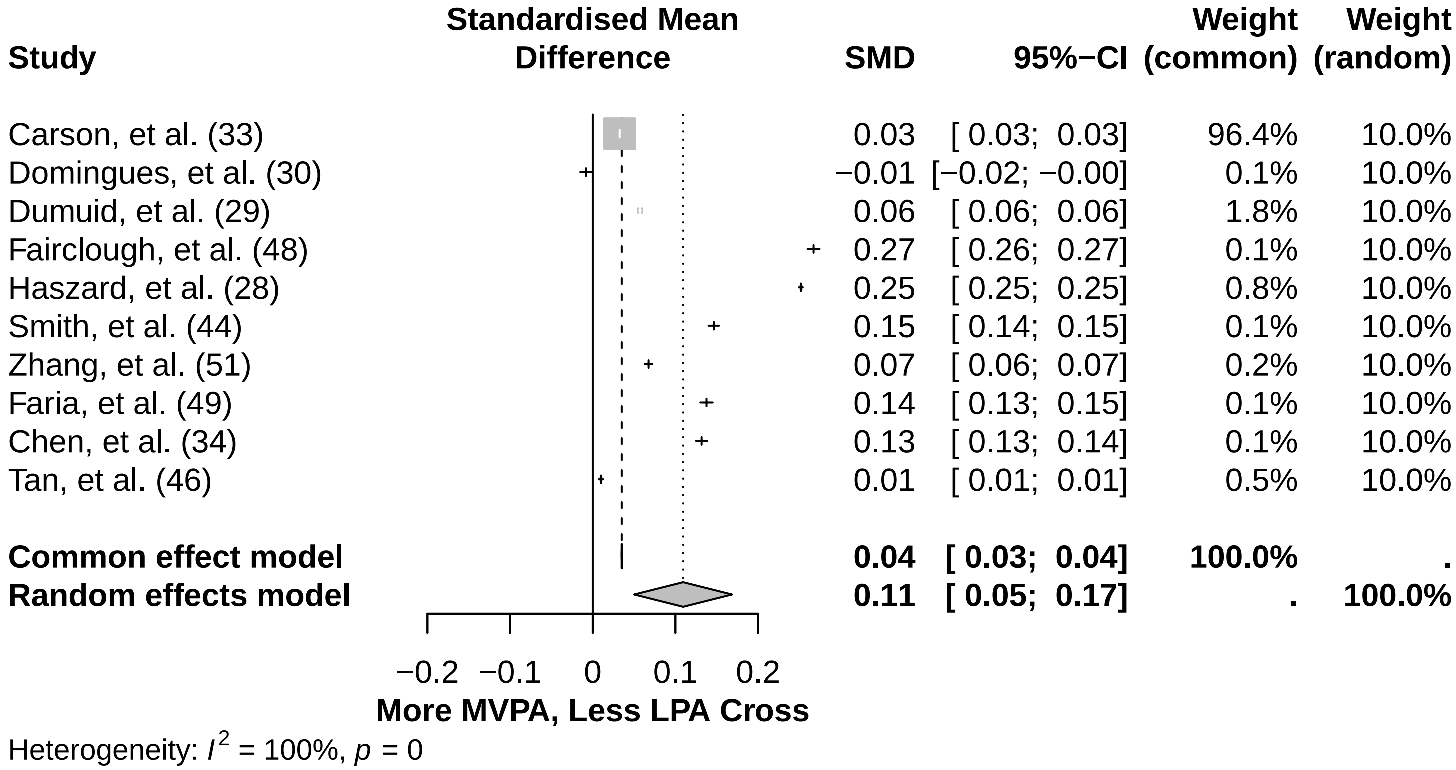
Study
Standardised Mean Difference
SMD
95%−CI
Weight (common)
Weight (random)
Carson, et al. (33) 0.03 [0.03; 0.03] 96.4% 10.0%
Domingues, et al. (30) −0.01 [−0.02; −0.00] 0.1% 10.0%
Dumuid, et al. (29) 0.06 [0.06; 0.06] 1.8% 10.0%
Fairclough, et al. (48) 0.27 [0.26; 0.27] 0.1% 10.0%
Haszard, et al. (28) 0.25 [0.25; 0.25] 0.8% 10.0%
Smith, et al. (44) 0.15 [0.14; 0.15] 0.1% 10.0%
Zhang, et al. (51) 0.07 [0.06; 0.07] 0.2% 10.0%
Faria, et al. (49) 0.14 [0.13; 0.15] 0.1% 10.0%
Chen, et al. (34) 0.13 [0.13; 0.14] 0.1% 10.0%
Tan, et al. (46) 0.01 [0.01; 0.01] 0.5% 10.0%
Common effect model 0.04 [0.03; 0.04] 100.0% .
Random effects model 0.11 [0.05; 0.17] . 100.0%
−0.2 −0.1 0 0.1 0.2
More MVPA, Less LPA Cross
Heterogeneity: I2 = 100%, p = 0

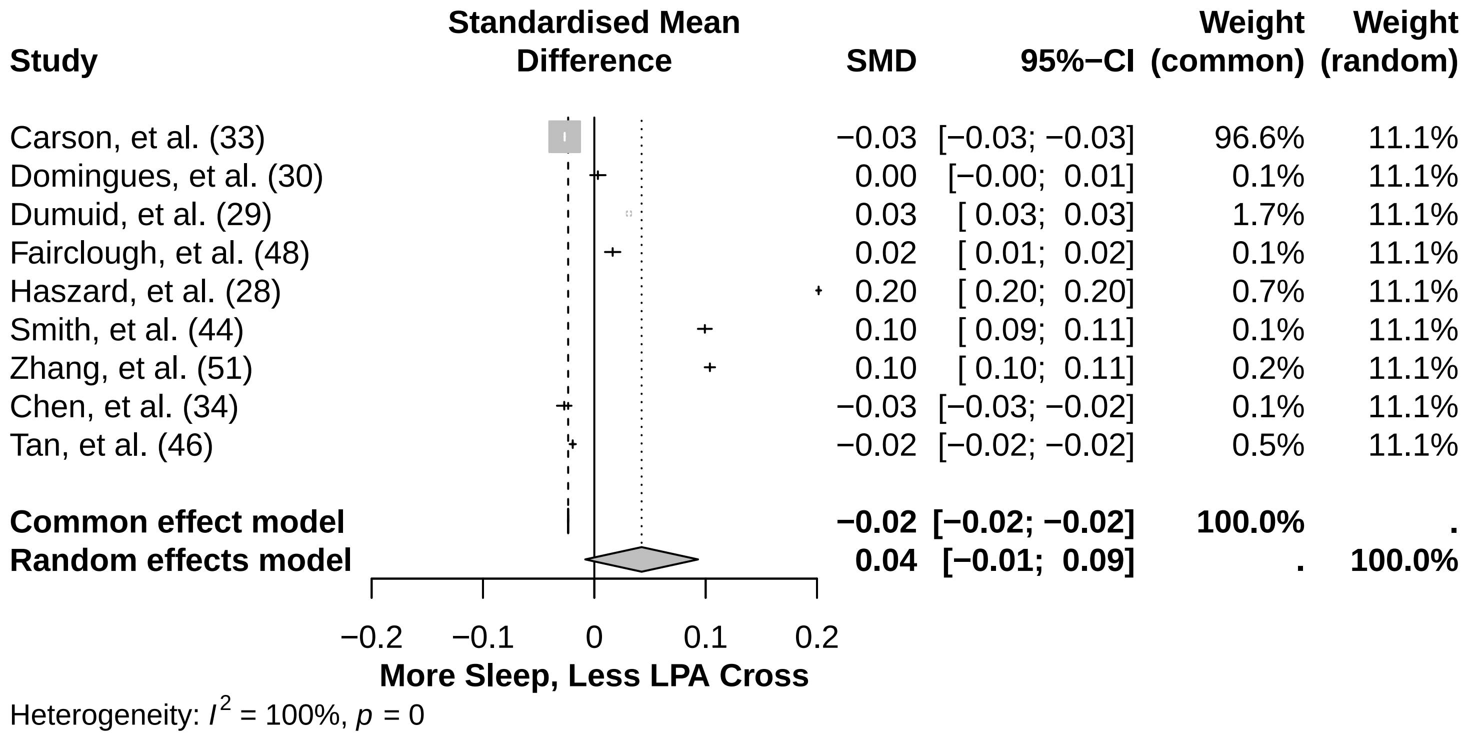
Study
Standardised Mean Difference
SMD
95%−CI
Weight (common)
Weight (random)
Carson, et al. (33) −0.03 [−0.03; −0.03] 96.6% 11.1%
Domingues, et al. (30) 0.00 [−0.00; 0.01] 0.1% 11.1%
Dumuid, et al. (29) 0.03 [0.03; 0.03] 1.7% 11.1%
Fairclough, et al. (48) 0.02 [0.01; 0.02] 0.1% 11.1%
Haszard, et al. (28) 0.20 [0.20; 0.20] 0.7% 11.1%
Smith, et al. (44) 0.10 [0.09; 0.11] 0.1% 11.1%
Zhang, et al. (51) 0.10 [0.10; 0.11] 0.2% 11.1%
Chen, et al. (34) −0.03 [−0.03; −0.02] 0.1% 11.1%
Tan, et al. (46) −0.02 [−0.02; −0.02] 0.5% 11.1%
Common effect model −0.02 [−0.02; −0.02] 100.0% .
Random effects model 0.04 [−0.01; 0.09] . 100.0%
−0.2
−0.1
0
0.1
0.2
More Sleep, Less LPA Cross
Heterogeneity: I2 = 100%, p = 0

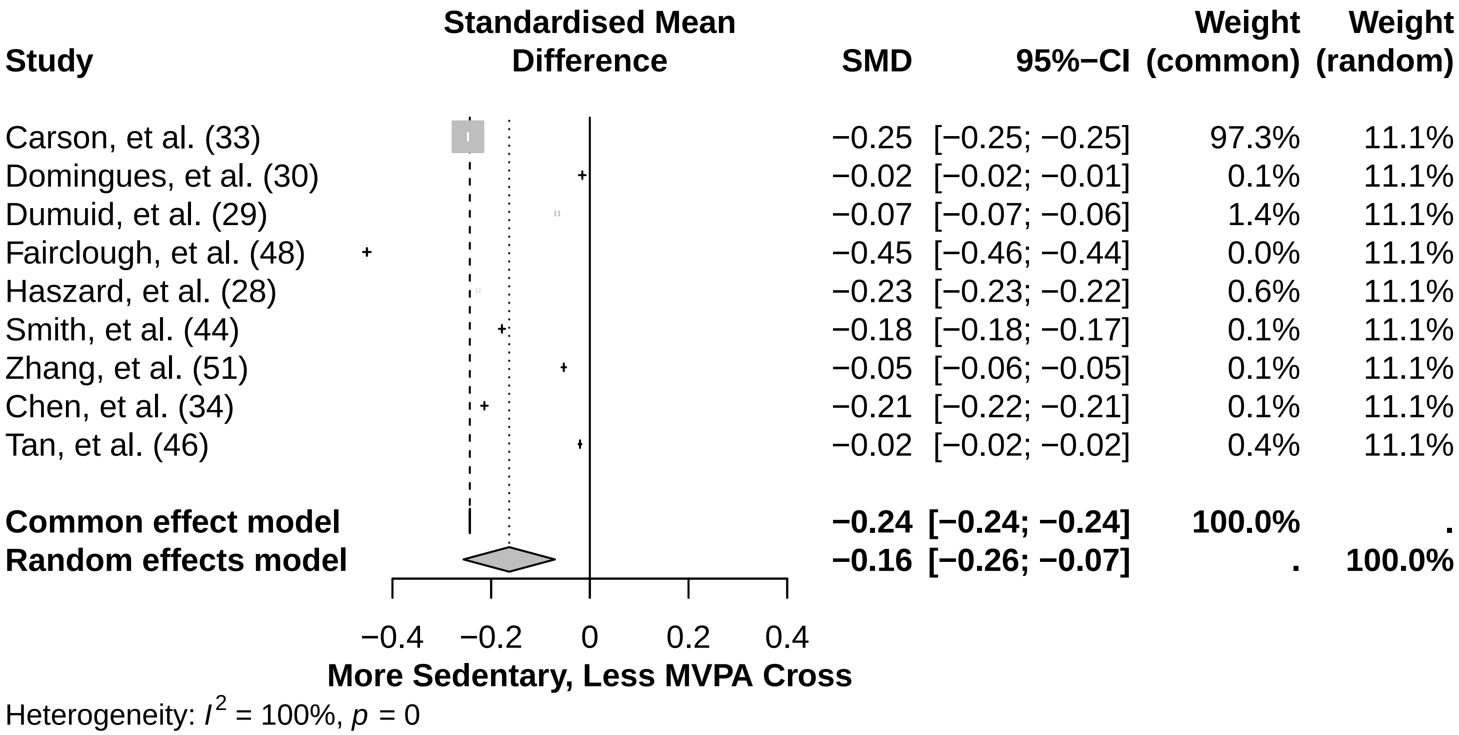
Study
Standardised Mean Difference
SMD
95%−CI
Weight (common)
Weight (random)
Carson, et al. (33) −0.25 [−0.25; −0.25] 97.3% 11.1%
Domingues, et al. (30) −0.02 [−0.02; −0.01] 0.1% 11.1%
Dumuid, et al. (29) −0.07 [−0.07; −0.06] 1.4% 11.1%
Fairclough, et al. (48) −0.45 [−0.46; −0.44] 0.0% 11.1%
Haszard, et al. (28) −0.23 [−0.23; −0.22] 0.6% 11.1%
Smith, et al. (44) −0.18 [−0.18; −0.17] 0.1% 11.1%
Zhang, et al. (51) −0.05 [−0.06; −0.05] 0.1% 11.1%
Chen, et al. (34) −0.21 [−0.22; −0.21] 0.1% 11.1%
Tan, et al. (46) −0.02 [−0.02; −0.02] 0.4% 11.1%
Common effect model −0.24 [−0.24; −0.24] 100.0% .
Random effects model −0.16 [−0.26; −0.07] . 100.0%
−0.4
−0.2
0
0.2
0.4
More Sedentary, Less MVPA Cross
Heterogeneity: I^2 = 100%, p = 0

Study	SMD	95%−CI	Weight (common)	Weight (random)
Carson, et al. (33)	−0.34	[−0.34; −0.34]	96.9%	11.1%
Domingues, et al. (30)	−0.02	[−0.03; −0.01]	0.1%	11.1%
Dumuid, et al. (29)	−0.06	[−0.06; −0.06]	1.6%	11.1%
Fairclough, et al. (48)	−0.47	[−0.48; −0.47]	0.1%	11.1%
Haszard, et al. (28)	−0.24	[−0.24; −0.24]	0.7%	11.1%
Smith, et al. (44)	−0.14	[−0.14; −0.13]	0.1%	11.1%
Zhang, et al. (51)	−0.06	[−0.06; −0.05]	0.2%	11.1%
Chen, et al. (34)	−0.18	[−0.19; −0.17]	0.1%	11.1%
Tan, et al. (46)	−0.01	[−0.02; −0.01]	0.4%	11.1%
**Common effect model**	**−0.33**	**[−0.33; −0.33]**	**100.0%**	**.**
**Random effects model**	**−0.17**	**[−0.27; −0.07]**	**.**	**100.0%**

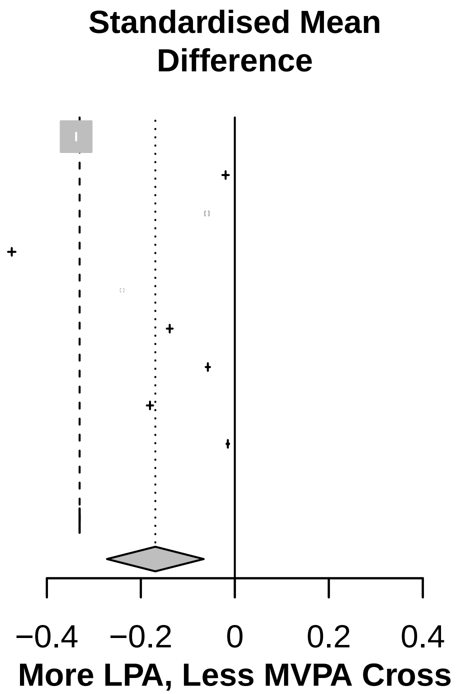

Heterogeneity: $I^2 = 100\%$, $p = 0$

Study	SMD	95%−CI	Weight (common)	Weight (random)
Carson, et al. (33)	−0.55	[−0.55; −0.55]	98.0%	11.1%
Domingues, et al. (30)	−0.02	[−0.02; −0.01]	0.0%	11.1%
Dumuid, et al. (29)	−0.05	[−0.06; −0.05]	1.0%	11.1%
Fairclough, et al. (48)	−0.46	[−0.47; −0.46]	0.0%	11.1%
Haszard, et al. (28)	−0.14	[−0.15; −0.14]	0.4%	11.1%
Smith, et al. (44)	0.01	[0.01; 0.02]	0.1%	11.1%
Zhang, et al. (51)	−0.05	[−0.05; −0.04]	0.1%	11.1%
Chen, et al. (34)	−0.21	[−0.21; −0.20]	0.0%	11.1%
Tan, et al. (46)	−0.01	[−0.01; −0.01]	0.3%	11.1%
**Common effect model**	**−0.54**	**[−0.54; −0.54]**	**100.0%**	**.**
**Random effects model**	**−0.16**	**[−0.30; −0.03]**	**.**	**100.0%**

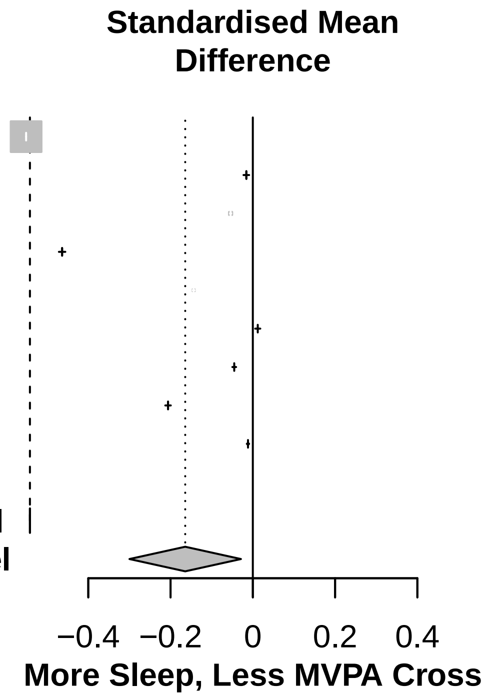

Heterogeneity: $I^2 = 100\%$, $p = 0$

Study	Standardised Mean Difference	SMD	95%−CI	Weight (common)	Weight (random)
Carson, et al. (33)		−0.01	[−0.01; −0.00]	96.4%	11.1%
Domingues, et al. (30)		0.00	[−0.00; 0.01]	0.1%	11.1%
Dumuid, et al. (29)		−0.03	[−0.03; −0.03]	1.8%	11.1%
Fairclough, et al. (48)		0.01	[0.01; 0.02]	0.1%	11.1%
Haszard, et al. (28)		−0.11	[−0.11; −0.11]	0.8%	11.1%
Smith, et al. (44)		−0.13	[−0.13; −0.12]	0.1%	11.1%
Zhang, et al. (51)		−0.06	[−0.07; −0.06]	0.2%	11.1%
Chen, et al. (34)		−0.00	[−0.01; 0.00]	0.1%	11.1%
Tan, et al. (46)		0.01	[0.00; 0.01]	0.5%	11.1%
**Common effect model**		**−0.01**	**[−0.01; −0.01]**	**100.0%**	**.**
**Random effects model**		**−0.04**	**[−0.07; −0.00]**	**.**	**100.0%**

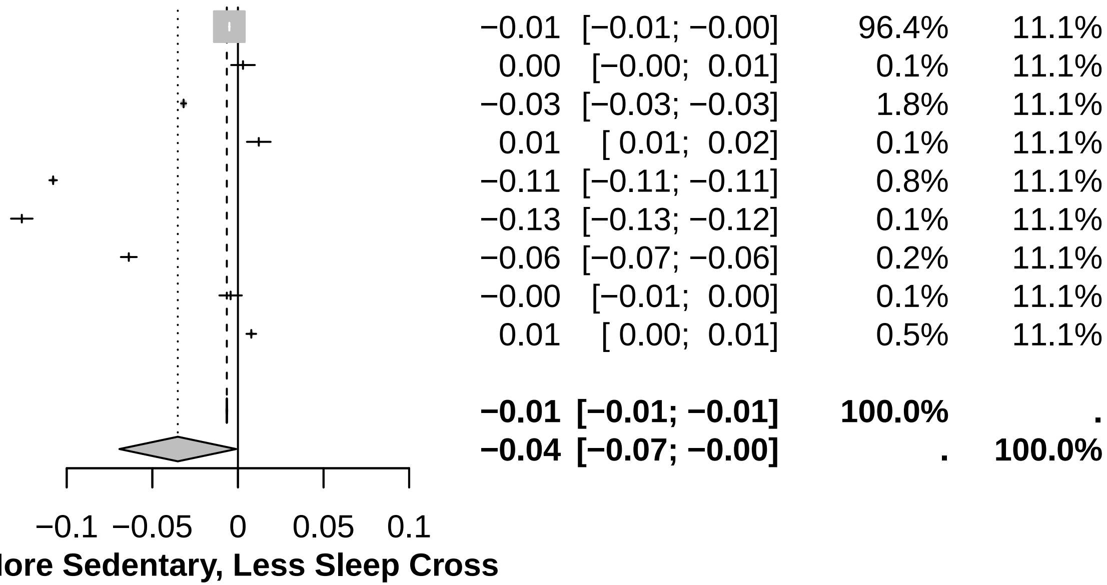

Heterogeneity: $I^2 = 100\%$, $p = 0$

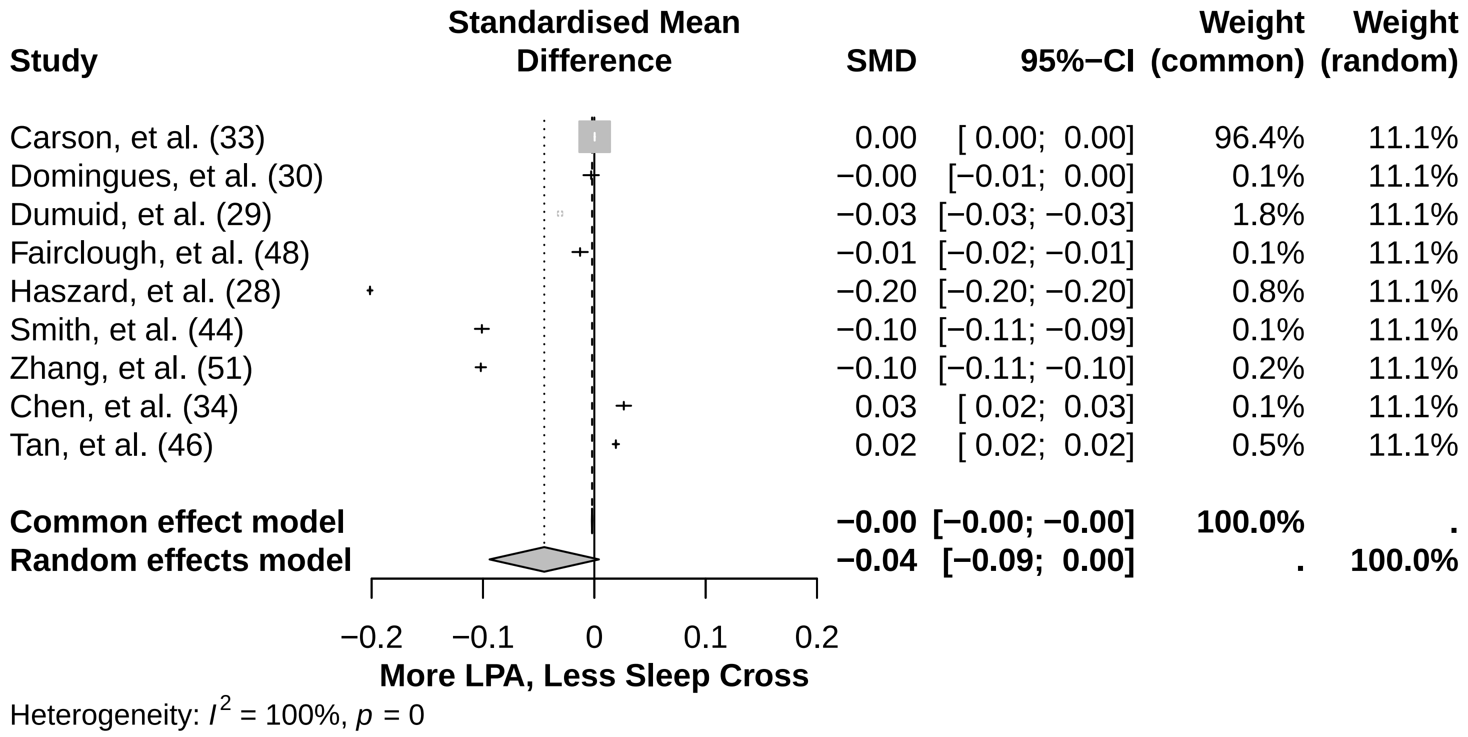
Study
Standardised Mean Difference
SMD
95%−CI
Weight (common)
Weight (random)
Carson, et al. (33) 0.00 [0.00; 0.00] 96.4% 11.1%
Domingues, et al. (30) −0.00 [−0.01; 0.00] 0.1% 11.1%
Dumuid, et al. (29) −0.03 [−0.03; −0.03] 1.8% 11.1%
Fairclough, et al. (48) −0.01 [−0.02; −0.01] 0.1% 11.1%
Haszard, et al. (28) −0.20 [−0.20; −0.20] 0.8% 11.1%
Smith, et al. (44) −0.10 [−0.11; −0.09] 0.1% 11.1%
Zhang, et al. (51) −0.10 [−0.11; −0.10] 0.2% 11.1%
Chen, et al. (34) 0.03 [0.02; 0.03] 0.1% 11.1%
Tan, et al. (46) 0.02 [0.02; 0.02] 0.5% 11.1%
Common effect model −0.00 [−0.00; −0.00] 100.0% .
Random effects model −0.04 [−0.09; 0.00] . 100.0%
−0.2 −0.1 0 0.1 0.2
More LPA, Less Sleep Cross
Heterogeneity: I2 = 100%, p = 0

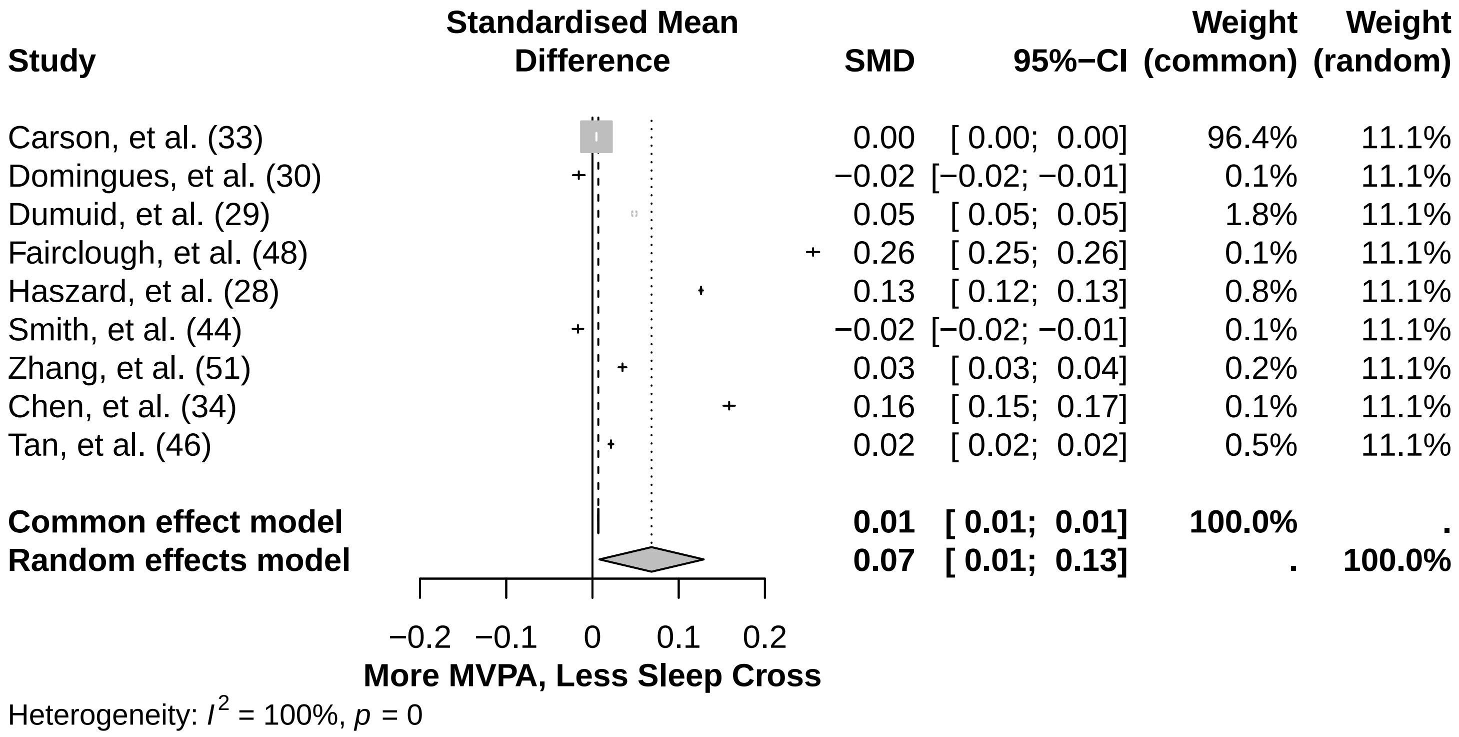
Study
Standardised Mean Difference
SMD
95%−CI
Weight (common)
Weight (random)
Carson, et al. (33) 0.00 [0.00; 0.00] 96.4% 11.1%
Domingues, et al. (30) −0.02 [−0.02; −0.01] 0.1% 11.1%
Dumuid, et al. (29) 0.05 [0.05; 0.05] 1.8% 11.1%
Fairclough, et al. (48) 0.26 [0.25; 0.26] 0.1% 11.1%
Haszard, et al. (28) 0.13 [0.12; 0.13] 0.8% 11.1%
Smith, et al. (44) −0.02 [−0.02; −0.01] 0.1% 11.1%
Zhang, et al. (51) 0.03 [0.03; 0.04] 0.2% 11.1%
Chen, et al. (34) 0.16 [0.15; 0.17] 0.1% 11.1%
Tan, et al. (46) 0.02 [0.02; 0.02] 0.5% 11.1%
Common effect model 0.01 [0.01; 0.01] 100.0% .
Random effects model 0.07 [0.01; 0.13] . 100.0%
−0.2
−0.1
0
0.1
0.2
More MVPA, Less Sleep Cross
Heterogeneity: I2 = 100%, p = 0

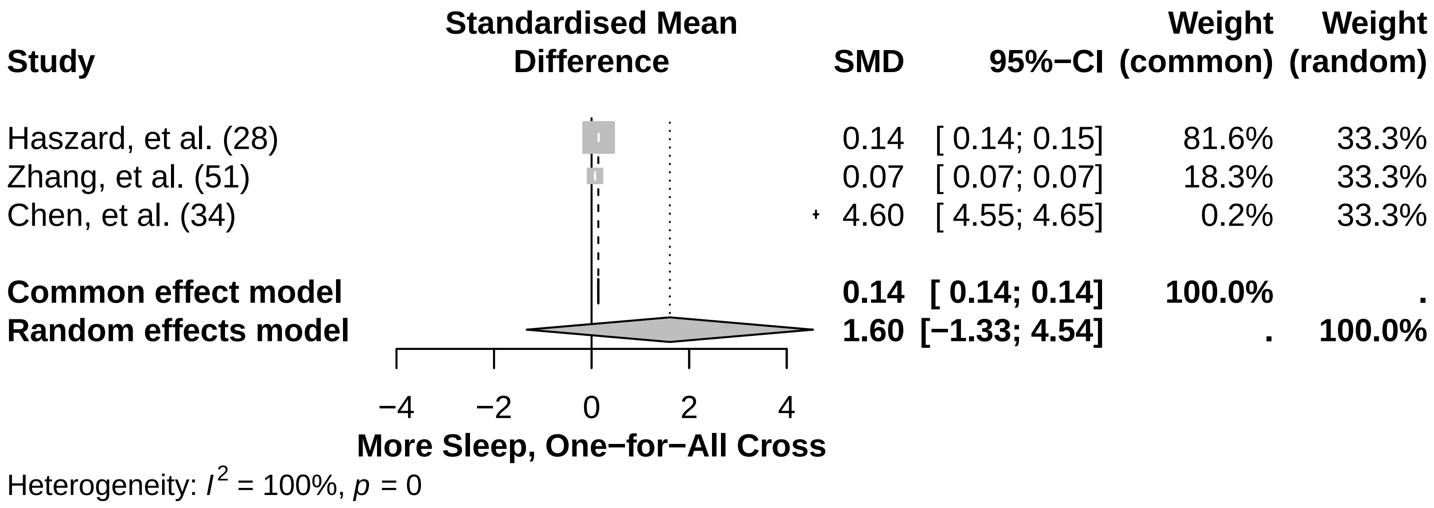
Study
Standardised Mean Difference
SMD
95%−CI
Weight (common)
Weight (random)
Haszard, et al. (28) 0.14 [0.14; 0.15] 81.6% 33.3%
Zhang, et al. (51) 0.07 [0.07; 0.07] 18.3% 33.3%
Chen, et al. (34) 4.60 [4.55; 4.65] 0.2% 33.3%
Common effect model 0.14 [0.14; 0.14] 100.0% .
Random effects model 1.60 [−1.33; 4.54] . 100.0%
−4 −2 0 2 4
More Sleep, One−for−All Cross
Heterogeneity: I2 = 100%, p = 0

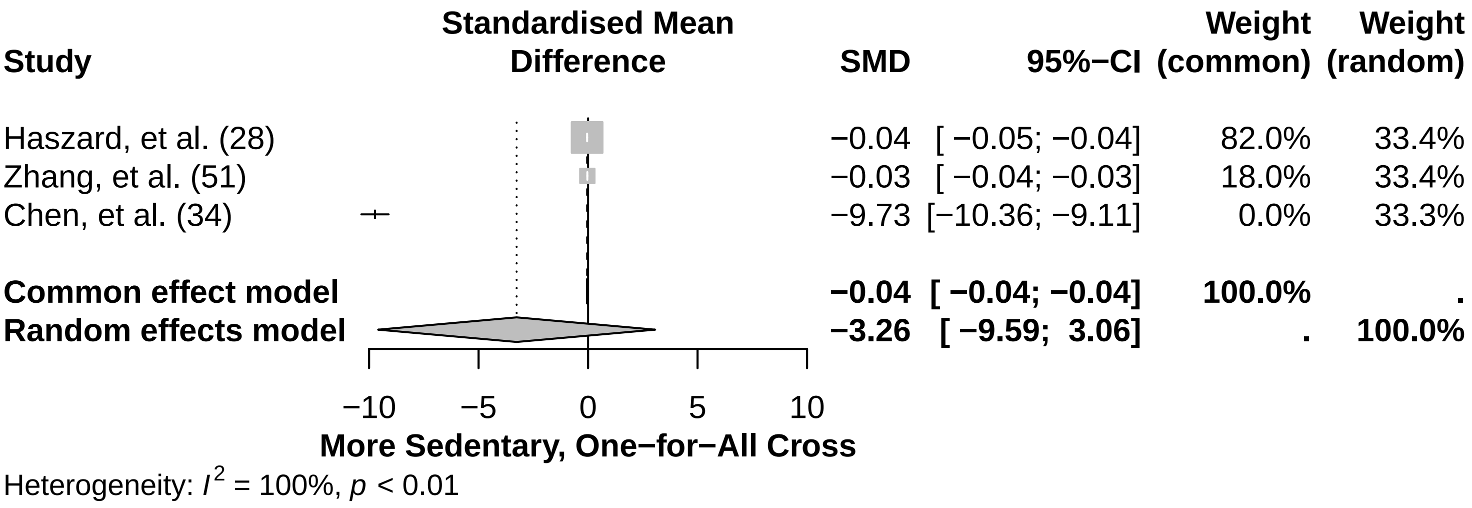

Standardised Mean Difference
Study
SMD
95%−CI
Weight (common)
Weight (random)
Haszard, et al. (28) −0.04 [−0.05; −0.04] 82.0% 33.4%
Zhang, et al. (51) −0.03 [−0.04; −0.03] 18.0% 33.4%
Chen, et al. (34) −9.73 [−10.36; −9.11] 0.0% 33.3%
Common effect model −0.04 [−0.04; −0.04] 100.0% .
Random effects model −3.26 [−9.59; 3.06] . 100.0%
−10
−5
0
5
10
More Sedentary, One−for−All Cross
Heterogeneity: I^2 = 100%, p < 0.01

Study	SMD	95%−CI	Weight (common)	Weight (random)
Haszard, et al. (28)	−0.22	[−0.22; −0.21]	81.2%	33.3%
Zhang, et al. (51)	−0.08	[−0.09; −0.08]	18.7%	33.3%
Chen, et al. (34)	3.79	[3.70; 3.89]	0.0%	33.3%
**Common effect model**	**−0.19**	**[−0.19; −0.19]**	**100.0%**	**.**
**Random effects model**	**1.16**	**[−1.41; 3.74]**	**.**	**100.0%**

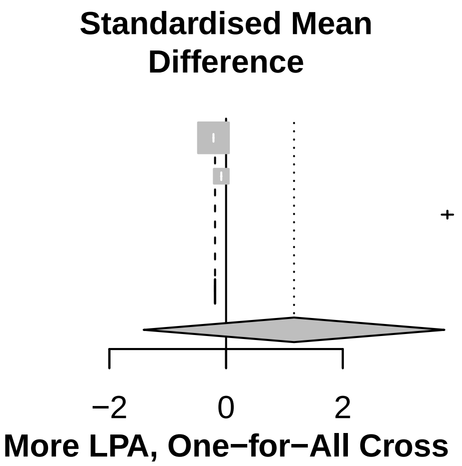

Heterogeneity: I^2 = 100%, p = 0

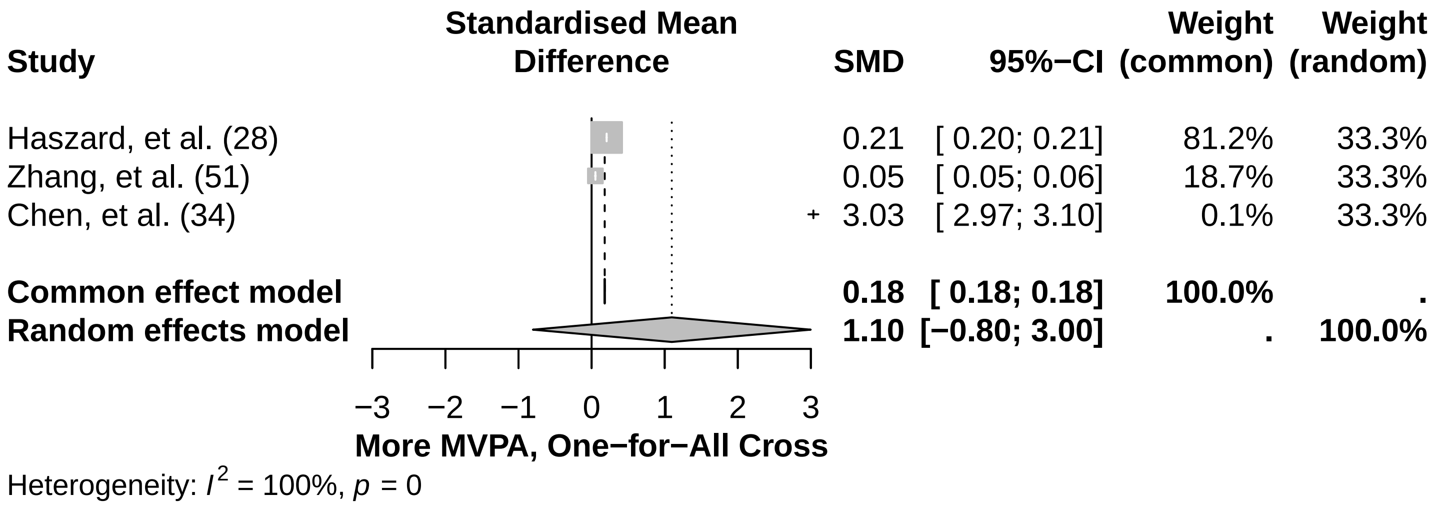
Study
Standardised Mean Difference
SMD
95%−CI
Weight (common)
Weight (random)
Haszard, et al. (28) 0.21 [0.20; 0.21] 81.2% 33.3%
Zhang, et al. (51) 0.05 [0.05; 0.06] 18.7% 33.3%
Chen, et al. (34) 3.03 [2.97; 3.10] 0.1% 33.3%
Common effect model 0.18 [0.18; 0.18] 100.0% .
Random effects model 1.10 [−0.80; 3.00] . 100.0%
−3 −2 −1 0 1 2 3
More MVPA, One−for−All Cross
Heterogeneity: I2 = 100%, p = 0

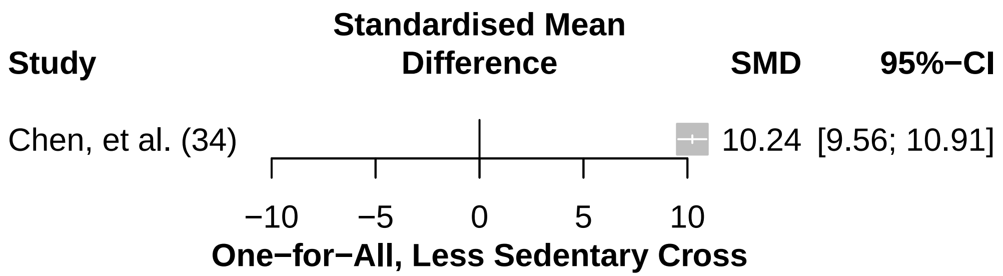
Study
Standardised Mean Difference
SMD
95%−CI
Chen, et al. (34)
10.24
[9.56; 10.91]
−10
−5
0
5
10
One−for−All, Less Sedentary Cross

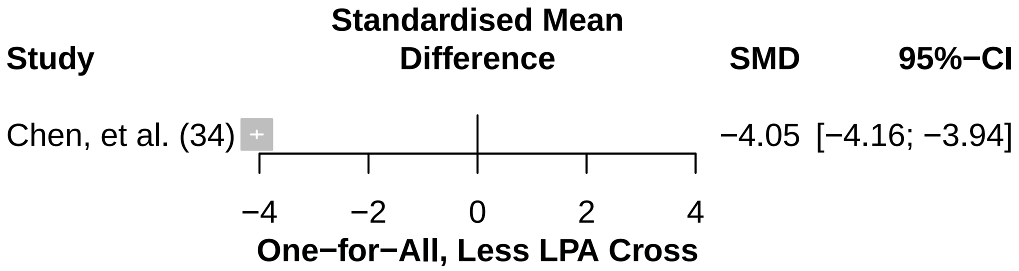
Study
Standardised Mean
Difference
SMD
95%−CI
Chen, et al. (34)
−4.05
[−4.16; −3.94]
−4
−2
0
2
4
One−for−All, Less LPA Cross

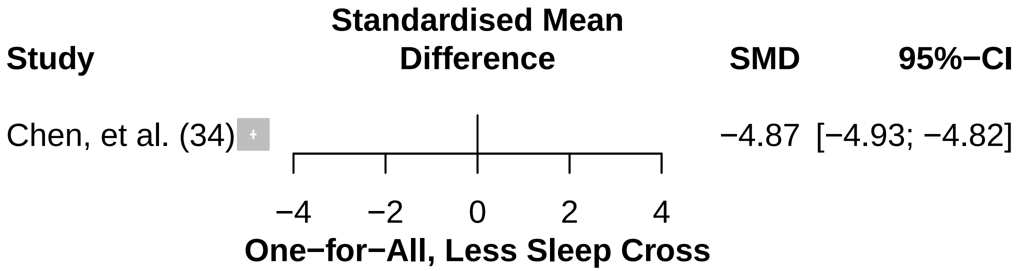
Study
Standardised Mean Difference
SMD
95%−CI
Chen, et al. (34)
−4.87
[−4.93; −4.82]
−4
−2
0
2
4
One−for−All, Less Sleep Cross

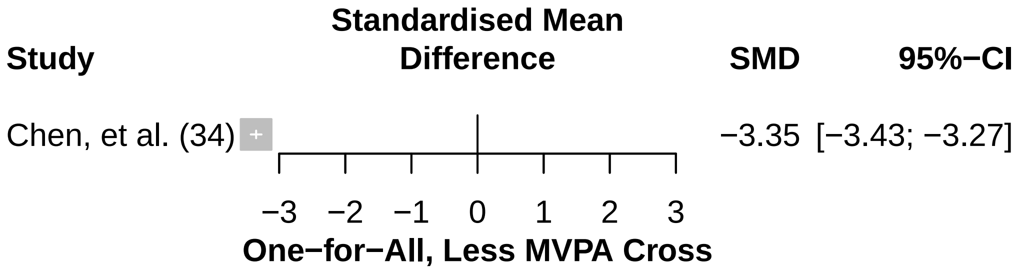

Study
Standardised Mean Difference
SMD
95%−CI
Chen, et al. (34)
−3.35
[−3.43; −3.27]
−3
−2
−1
0
1
2
3
One−for−All, Less MVPA Cross

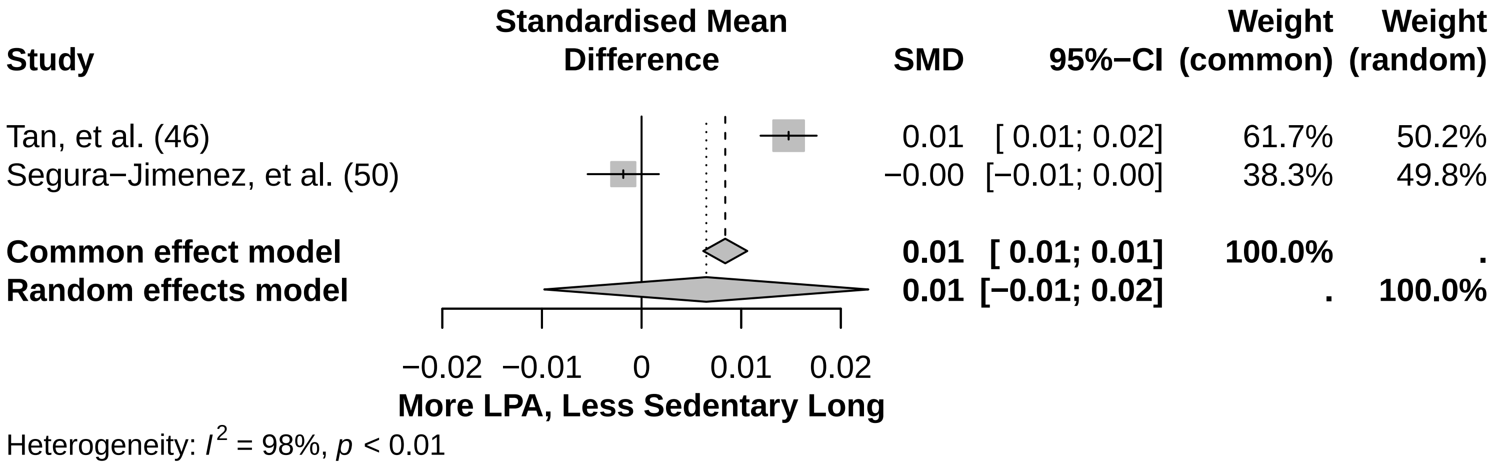
Study
Standardised Mean Difference
SMD
95%−CI
Weight (common)
Weight (random)
Tan, et al. (46) 0.01 [0.01; 0.02] 61.7% 50.2%
Segura−Jimenez, et al. (50) −0.00 [−0.01; 0.00] 38.3% 49.8%
Common effect model 0.01 [0.01; 0.01] 100.0% .
Random effects model 0.01 [−0.01; 0.02] . 100.0%
−0.02 −0.01 0 0.01 0.02
More LPA, Less Sedentary Long
Heterogeneity: I2 = 98%, p < 0.01

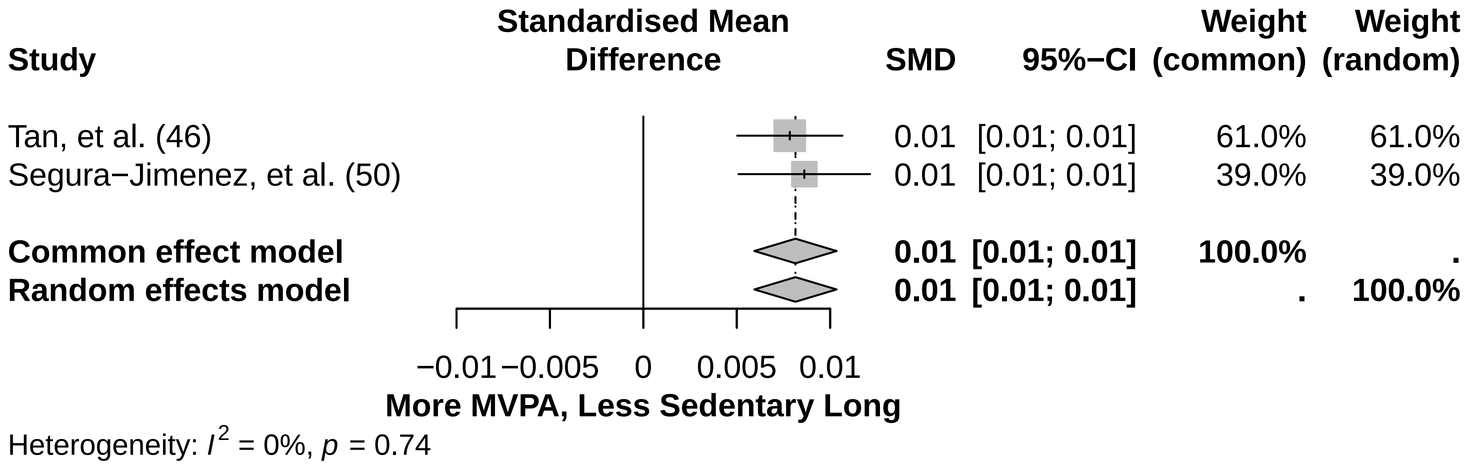
Study
Standardised Mean
Difference
SMD
95%−CI
Weight
(common)
Weight
(random)
Tan, et al. (46)
0.01
[0.01; 0.01]
61.0%
61.0%
Segura−Jimenez, et al. (50)
0.01
[0.01; 0.01]
39.0%
39.0%
Common effect model
0.01
[0.01; 0.01]
100.0%
.
Random effects model
0.01
[0.01; 0.01]
.
100.0%
−0.01
−0.005
0
0.005
0.01
More MVPA, Less Sedentary Long
Heterogeneity: I2 = 0%, p = 0.74

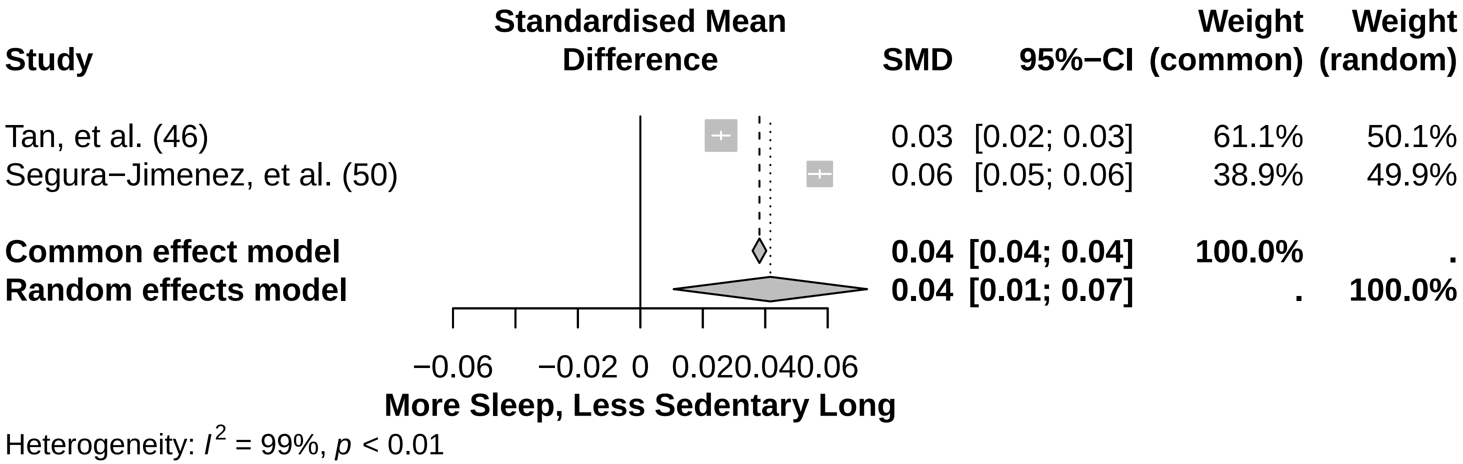
Study
Standardised Mean Difference
SMD
95%−CI
Weight (common)
Weight (random)
Tan, et al. (46) 0.03 [0.02; 0.03] 61.1% 50.1%
Segura−Jimenez, et al. (50) 0.06 [0.05; 0.06] 38.9% 49.9%
Common effect model 0.04 [0.04; 0.04] 100.0% .
Random effects model 0.04 [0.01; 0.07] . 100.0%
−0.06
−0.02
0
0.02
0.04
0.06
More Sleep, Less Sedentary Long
Heterogeneity: I2 = 99%, p < 0.01

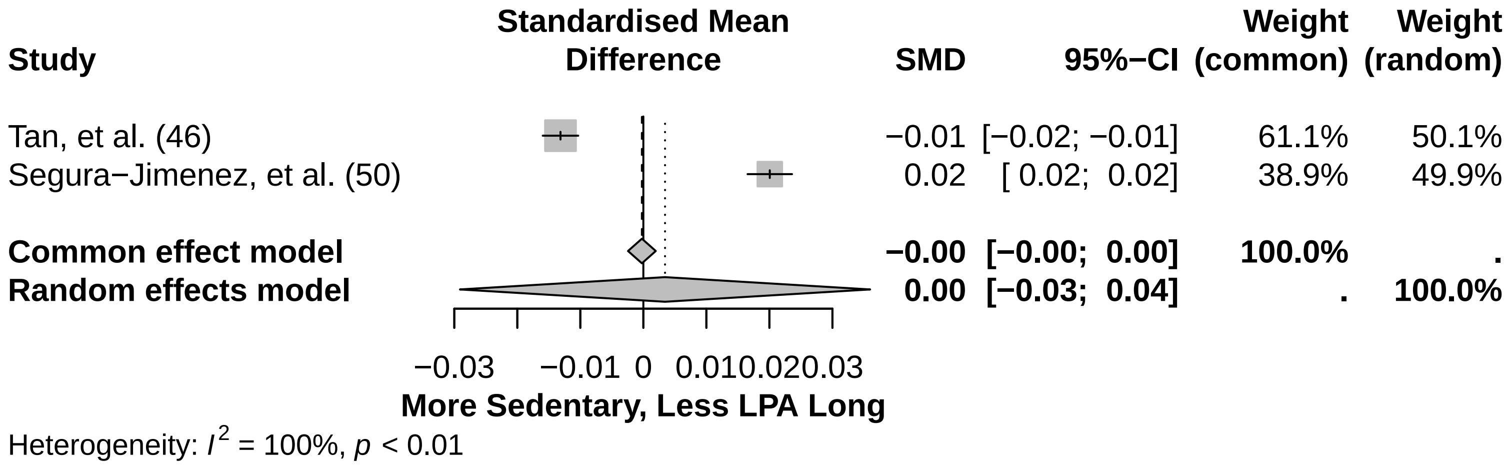
Study
Standardised Mean Difference
SMD
95%−CI
Weight (common)
Weight (random)
Tan, et al. (46) −0.01 [−0.02; −0.01] 61.1% 50.1%
Segura−Jimenez, et al. (50) 0.02 [0.02; 0.02] 38.9% 49.9%
Common effect model −0.00 [−0.00; 0.00] 100.0% .
Random effects model 0.00 [−0.03; 0.04] . 100.0%
−0.03 −0.01 0 0.01 0.02 0.03
More Sedentary, Less LPA Long
Heterogeneity: I2 = 100%, p < 0.01

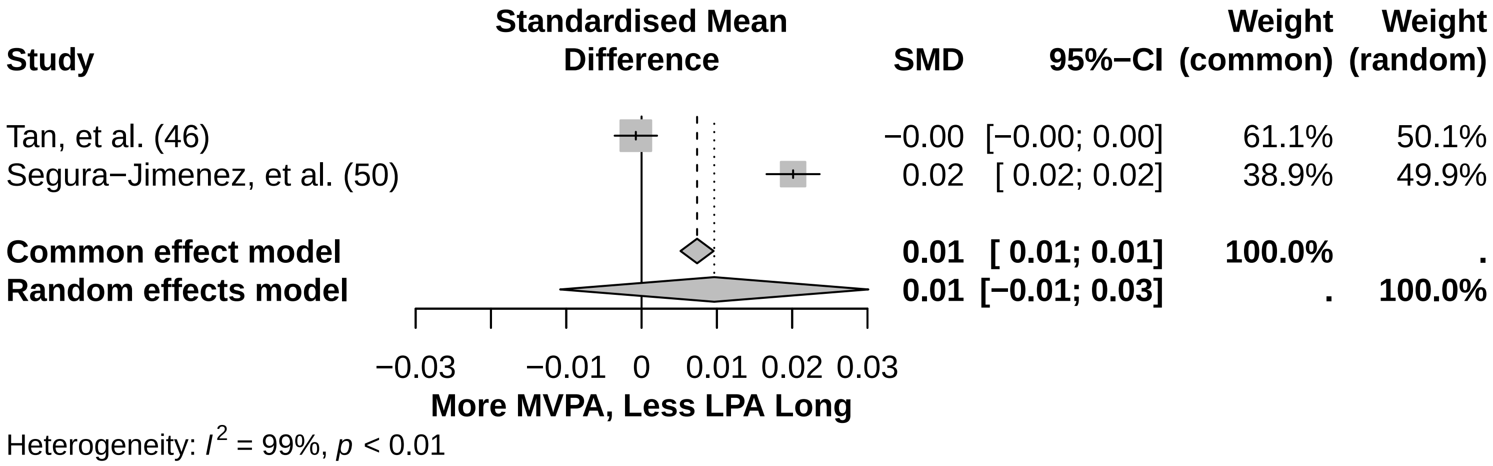
Study
Standardised Mean Difference
SMD
95%−CI
Weight (common)
Weight (random)
Tan, et al. (46) −0.00 [−0.00; 0.00] 61.1% 50.1%
Segura−Jimenez, et al. (50) 0.02 [0.02; 0.02] 38.9% 49.9%
Common effect model 0.01 [0.01; 0.01] 100.0% .
Random effects model 0.01 [−0.01; 0.03] . 100.0%
−0.03 −0.01 0 0.01 0.02 0.03
More MVPA, Less LPA Long
Heterogeneity: I2 = 99%, p < 0.01

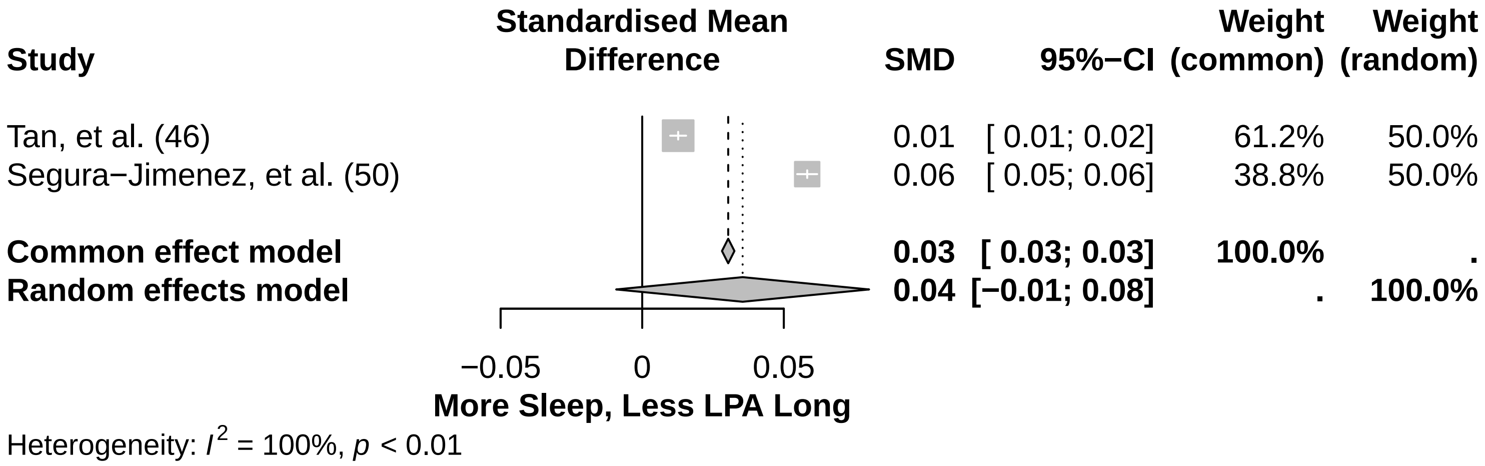

Study
Standardised Mean Difference
SMD
95%−CI
Weight (common)
Weight (random)
Tan, et al. (46) 0.01 [0.01; 0.02] 61.2% 50.0%
Segura−Jimenez, et al. (50) 0.06 [0.05; 0.06] 38.8% 50.0%
Common effect model 0.03 [0.03; 0.03] 100.0% .
Random effects model 0.04 [−0.01; 0.08] . 100.0%
−0.05
0
0.05
More Sleep, Less LPA Long
Heterogeneity: I^2 = 100%, p < 0.01

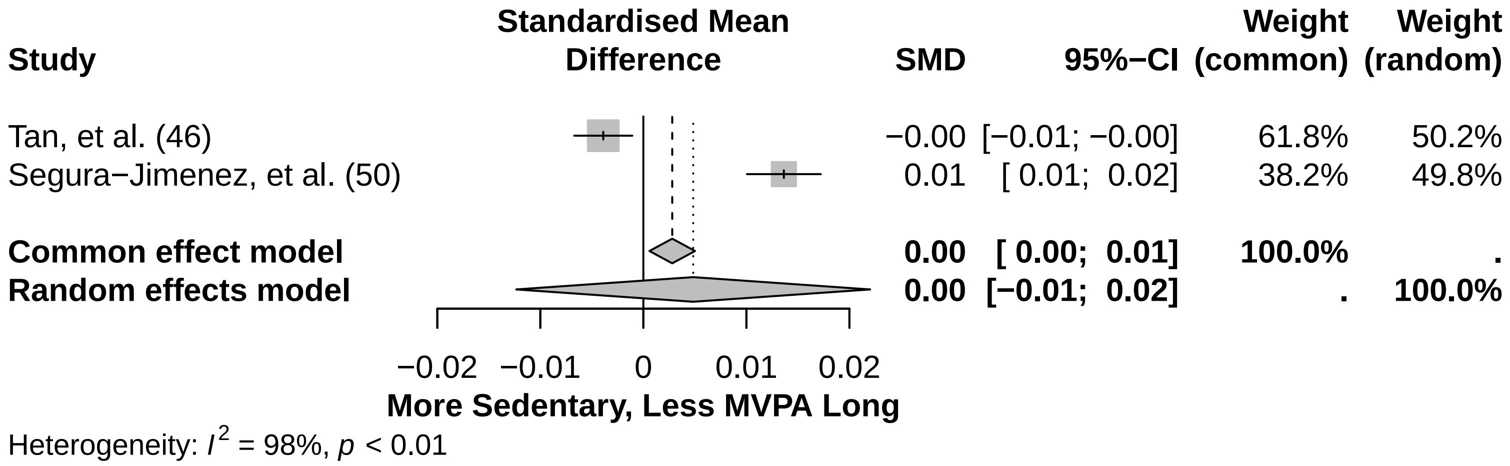
Study
Standardised Mean Difference
SMD
95%−CI
Weight (common)
Weight (random)
Tan, et al. (46) −0.00 [−0.01; −0.00] 61.8% 50.2%
Segura−Jimenez, et al. (50) 0.01 [0.01; 0.02] 38.2% 49.8%
Common effect model 0.00 [0.00; 0.01] 100.0% .
Random effects model 0.00 [−0.01; 0.02] . 100.0%
−0.02 −0.01 0 0.01 0.02
More Sedentary, Less MVPA Long
Heterogeneity: I2 = 98%, p < 0.01

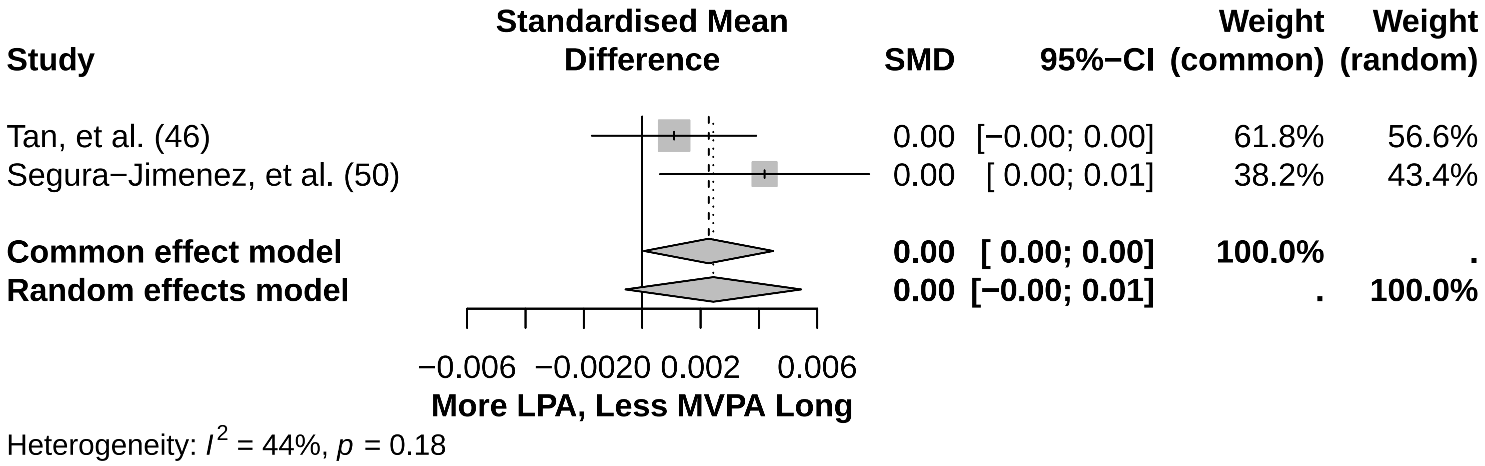
Study
Standardised Mean Difference
SMD
95%−CI
Weight (common)
Weight (random)
Tan, et al. (46) 0.00 [−0.00; 0.00] 61.8% 56.6%
Segura−Jimenez, et al. (50) 0.00 [0.00; 0.01] 38.2% 43.4%
Common effect model 0.00 [0.00; 0.00] 100.0% .
Random effects model 0.00 [−0.00; 0.01] . 100.0%
−0.006 −0.002 0 0.002 0.006
More LPA, Less MVPA Long
Heterogeneity: I^2 = 44%, p = 0.18

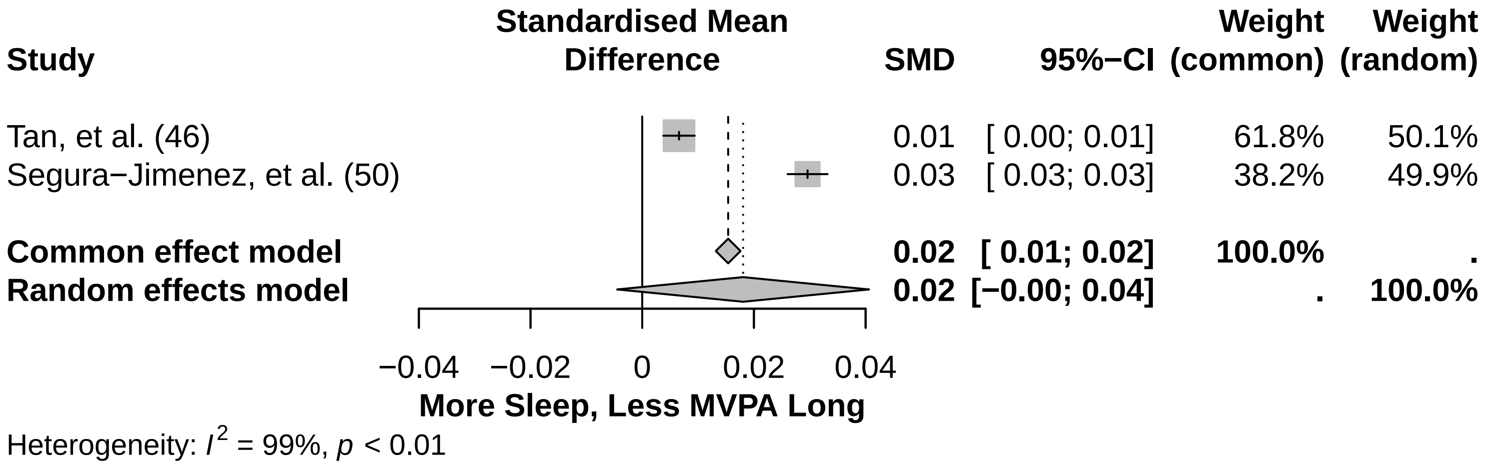

Study
Standardised Mean Difference
SMD
95%−CI
Weight (common)
Weight (random)
Tan, et al. (46) 0.01 [0.00; 0.01] 61.8% 50.1%
Segura−Jimenez, et al. (50) 0.03 [0.03; 0.03] 38.2% 49.9%
Common effect model 0.02 [0.01; 0.02] 100.0% .
Random effects model 0.02 [−0.00; 0.04] . 100.0%
−0.04 −0.02 0 0.02 0.04
More Sleep, Less MVPA Long
Heterogeneity: I2 = 99%, p < 0.01

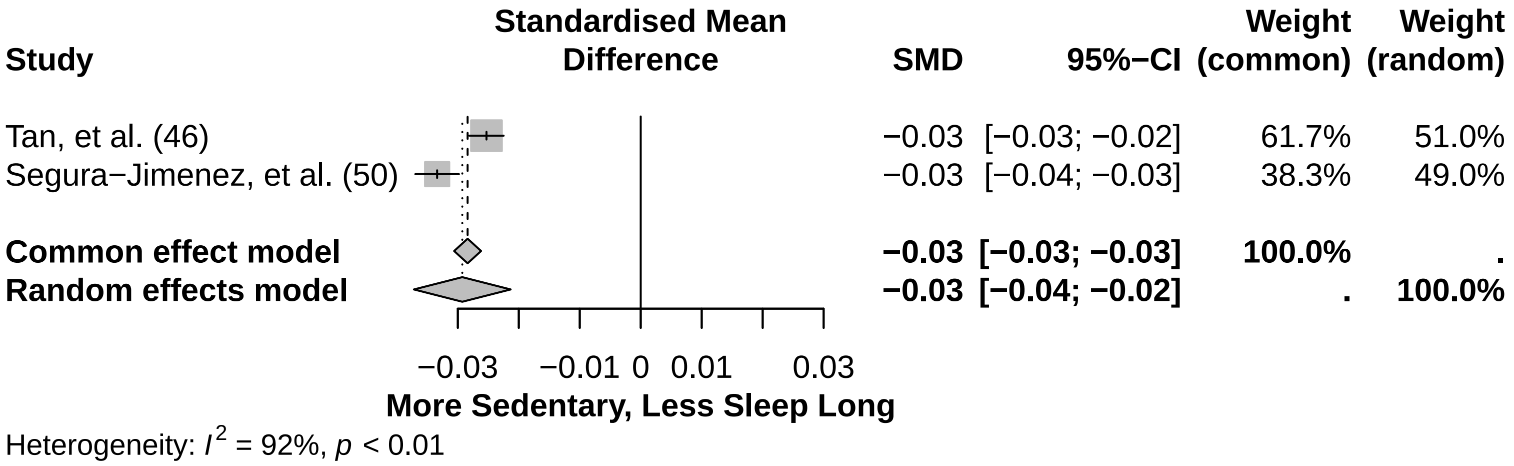
Study
Standardised Mean Difference
SMD
95%−CI
Weight (common)
Weight (random)
Tan, et al. (46)
−0.03 [−0.03; −0.02] 61.7% 51.0%
Segura−Jimenez, et al. (50)
−0.03 [−0.04; −0.03] 38.3% 49.0%
Common effect model
−0.03 [−0.03; −0.03] 100.0% .
Random effects model
−0.03 [−0.04; −0.02] . 100.0%
−0.03
−0.01
0
0.01
0.03
More Sedentary, Less Sleep Long
Heterogeneity: I2 = 92%, p < 0.01

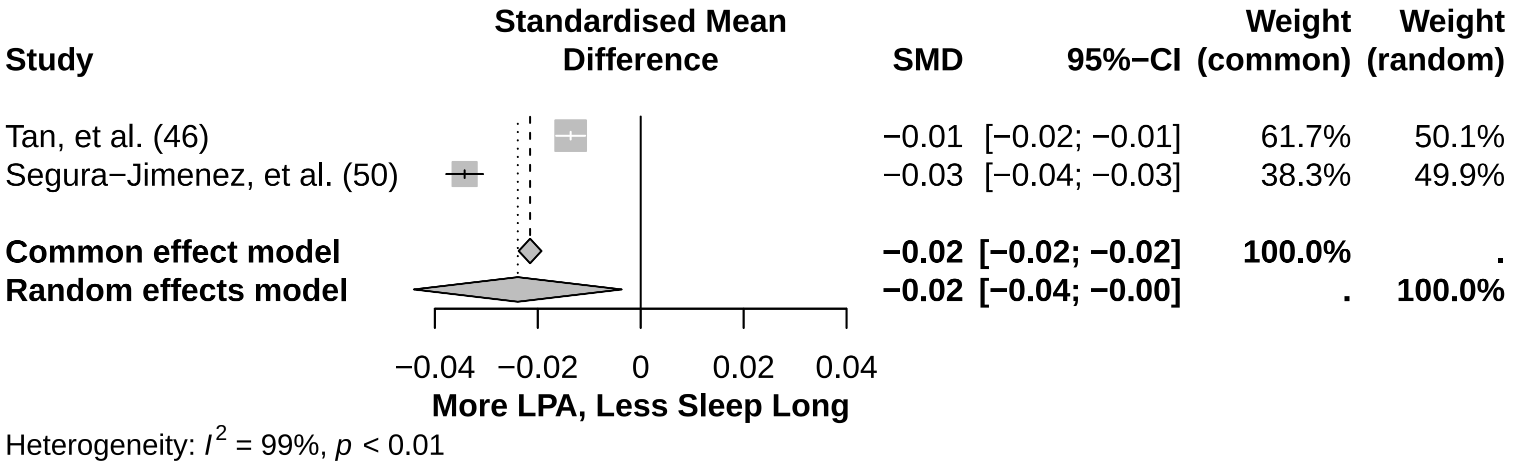
Study
Standardised Mean Difference
SMD
95%−CI
Weight (common)
Weight (random)
Tan, et al. (46) −0.01 [−0.02; −0.01] 61.7% 50.1%
Segura−Jimenez, et al. (50) −0.03 [−0.04; −0.03] 38.3% 49.9%
Common effect model −0.02 [−0.02; −0.02] 100.0% .
Random effects model −0.02 [−0.04; −0.00] . 100.0%
−0.04 −0.02 0 0.02 0.04
More LPA, Less Sleep Long
Heterogeneity: I2 = 99%, p < 0.01

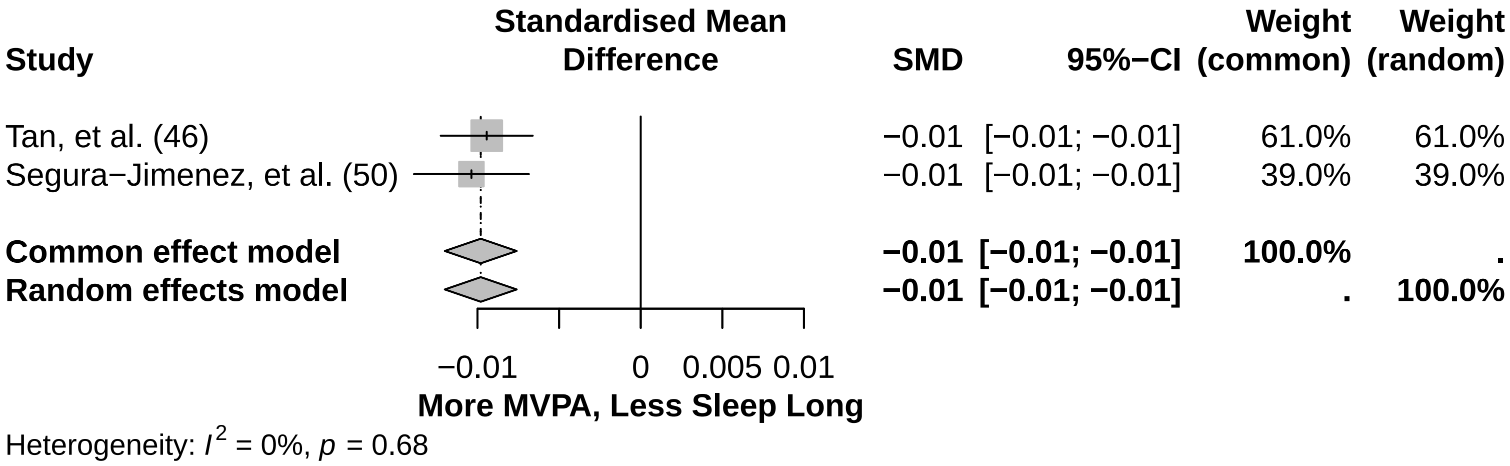
Study
Standardised Mean Difference
SMD
95%−CI
Weight (common)
Weight (random)
Tan, et al. (46) −0.01 [−0.01; −0.01] 61.0% 61.0%
Segura−Jimenez, et al. (50) −0.01 [−0.01; −0.01] 39.0% 39.0%
Common effect model −0.01 [−0.01; −0.01] 100.0% .
Random effects model −0.01 [−0.01; −0.01] . 100.0%
−0.01
0
0.005
0.01
More MVPA, Less Sleep Long
Heterogeneity: I2 = 0%, p = 0.68
