# Supplementary material for: A systematic review and meta-analysis of the first decade of compositional data analyses of 24-hour movement behaviours, health, and well-being in school-aged children
Source: J Act Sedentary Sleep Behav. 2025 Mar 27;4:4. doi: 10.1186/s44167-025-00076-w (PMC11948812; doi:10.1186/s44167-025-00076-w)
Supplement: Supplementary file 3 — Supplementary Material 3 [file 44167_2025_76_MOESM3_ESM.docx]

Ovid MEDLINE, EMBASE, and APA PsycINFO via Ovid; and CINAHL and SPORTDiscus via EBSCO were searched on May 24, 2024 using the base search strategy:

| **Step** | **Search** |
| --- | --- |
| 1 | (compositional data analys* or coda or compositional iso* or goldilocks) |
| 2 | physical activ* |
| 3 | sleep* |
| 4 | sedentary |
| 5 | (movement behaviour* or movement behavior* or 24-hour or 24 hour) |
| 6 | 2 or 3 or 4 or 5 |
| 7 | 1 and 6 |
