# Supplementary material for: A systematic review and meta-analysis of the first decade of compositional data analyses of 24-hour movement behaviours, health, and well-being in school-aged children
Source: J Act Sedentary Sleep Behav. 2025 Mar 27;4:4. doi: 10.1186/s44167-025-00076-w (PMC11948812; doi:10.1186/s44167-025-00076-w)
Supplement: Supplementary file 6 — Supplementary Material 6 [file 44167_2025_76_MOESM6_ESM.docx]

Additional File 4: Direction of associations for all compositional regression analyses

| **Health and Well-Being Categories** | **Exposure** | **F** | **U** | **N** | **Total** |
| --- | --- | --- | --- | --- | --- |
| Overall | Sedentary | 2 | 19 | **47** | 68 |
|  | LPA | 3 | 18 | **46** | 67 |
|  | MVPA | 23 | 0 | **42** | 65 |
|  | Sleep | 14 | 1 | **57** | 72 |
|  | TPA | 0 | 0 | 1 | 1 |
|  | Sedentary school | 0 | 0 | **4** | 4 |
|  | Sedentary out-of-school | 0 | **4** | 0 | 4 |
|  | LPA school | 0 | 0 | **4** | 4 |
|  | LPA out-of-school | 1 | 0 | **3** | 4 |
|  | MPA school | 0 | 0 | **4** | 4 |
|  | MPA out-of-school | 0 | 0 | **4** | 4 |
|  | VPA school | 0 | 0 | **4** | 4 |
|  | VPA out-of-school | 0 | 0 | **4** | 4 |
|  | MPA | 0 | 0 | **2** | 2 |
|  | VPA | 0 | 0 | **2** | 2 |
| Adiposity Indicators | Sedentary | 0 | **7** | 4 | 11 |
|  | LPA | 1 | **8** | 1 | 10 |
|  | MVPA | **10** | 0 | 0 | 10 |
|  | Sleep | 6 | 1 | **8** | 15 |
|  | TPA | 0 | 0 | 1 | 1 |
|  | Sedentary school | 0 | 0 | **4** | 4 |
|  | Sedentary out-of-school | 0 | **4** | 0 | 4 |
|  | LPA school | 0 | 0 | **4** | 4 |
|  | LPA out-of-school | 1 | 0 | **3** | 4 |
|  | MPA school | 0 | 0 | **4** | 4 |
|  | MPA out-of-school | 0 | 0 | **4** | 4 |
|  | VPA school | 0 | 0 | **4** | 4 |
|  | VPA out-of-school | 0 | 0 | **4** | 4 |
| Cardiometabolic Biomarkers | Sedentary | 0 | 2 | **9** | 11 |
|  | LPA | 0 | 1 | **10** | 11 |
|  | MVPA | **9** | 0 | 2 | 11 |
|  | Sleep | 2 | 0 | **9** | 11 |
| Cognitive Indicators | Sleep | 0 | 0 | **7** | 7 |
|  | Sedentary | 2 | 0 | **5** | 7 |
|  | LPA | 1 | **5** | 1 | 7 |
|  | MVPA | 0 | 0 | **7** | 7 |
| Fitness | Sedentary | 0 | 1 | 0 | 1 |
|  | LPA | 0 | 0 | 1 | 1 |
|  | MVPA | 1 | 0 | 0 | 1 |
|  | Sleep | 0 | 0 | 1 | 1 |
| Mental Well-Being and Illness Indicators* | Sedentary | 0 | 8 | **28** | 36 |
|  | LPA | 1 | 3 | **32** | 36 |
|  | MVPA | 2 | 0 | **32** | 34 |
|  | Sleep | 5 | 0 | **31** | 36 |
|  | MPA | 0 | 0 | **2** | 2 |
|  | VPA | 0 | 0 | **2** | 2 |
| Motor skills | Sleep | 0 | 0 | 1 | 1 |
|  | Sedentary | 0 | 0 | 1 | 1 |
|  | LPA | 0 | 0 | 1 | 1 |
|  | MVPA | 0 | 0 | 1 | 1 |
| Other Health Behaviours | Sleep | 1 | 0 | 0 | 1 |
|  | Sedentary | 0 | 1 | 0 | 1 |
|  | LPA | 0 | 1 | 0 | 1 |
|  | MVPA | 1 | 0 | 0 | 1 |

F = Favourable, U = Unfavourable, N = Null, LPA = light physical activity, MVPA = moderate to vigorous physical activity, TPA = total physical activity, MPA = moderate physical activity, VPA = vigorous physical activity, * = 12/36 associations are longitudinal from two studies, with all being null except for 3/12 unfavourable associations for sedentary behaviour. Bolded values indicate more than 50% of associations are in that direction, when more than one association is examined.
